# The host inflammatory response contributes to disease severity in Crimean-Congo hemorrhagic fever virus infected mice

**DOI:** 10.1371/journal.ppat.1010485

**Published:** 2022-05-19

**Authors:** Joseph W. Golden, Xiankun Zeng, Curtis R. Cline, Jeffrey M. Smith, Sharon P. Daye, Brian D. Carey, Candace D. Blancett, Charles J. Shoemaker, Jun Liu, Collin J. Fitzpatrick, Christopher P. Stefan, Aura R. Garrison

**Affiliations:** 1 Virology Division, United States Army Medical Research Institute of Infectious Diseases, Fort Detrick, Maryland, United States of America; 2 Pathology Division, United States Army Medical Research Institute of Infectious Diseases, Fort Detrick, Maryland, United States of America; 3 Diagnostic Services Division, United States Army Medical Research Institute of Infectious Diseases, Fort Detrick, Maryland, United States of America; CDC, UNITED STATES

## Abstract

Crimean-Congo hemorrhagic fever virus (CCHFV) is an important human pathogen. In cell culture, CCHFV is sensed by the cytoplasmic RNA sensor retinoic acid-inducible gene I (RIG-I) molecule and its adaptor molecule mitochondrial antiviral signaling (MAVS) protein. MAVS initiates both type I interferon (IFN-I) and proinflammatory responses. Here, we studied the role MAVS plays in CCHFV infection in mice in both the presence and absence of IFN-I activity. MAVS-deficient mice were not susceptible to CCHFV infection when IFN-I signaling was active and showed no signs of disease. When IFN-I signaling was blocked by antibody, MAVS-deficient mice lost significant weight, but were uniformly protected from lethal disease, whereas all control mice succumbed to infection. Cytokine activity in the infected MAVS-deficient mice was markedly blunted. Subsequent investigation revealed that CCHFV infected mice lacking TNF-α receptor signaling (TNFA-R-deficient), but not IL-6 or IL-1 activity, had more limited liver injury and were largely protected from lethal outcomes. Treatment of mice with an anti-TNF-α neutralizing antibody also conferred partial protection in a post-virus exposure setting. Additionally, we found that a disease causing, but non-lethal strain of CCHFV produced more blunted inflammatory cytokine responses compared to a lethal strain in mice. Our work reveals that MAVS activation and cytokine production both contribute to CCHFV pathogenesis, potentially identifying new therapeutic targets to treat this disease.

## Author summary

CCHFV causes a spectrum of disease in humans that can range from minimally symptomatic to a catastrophic and lethal infection. Factors dictating why some develop mild illness and others succumb to disease are unclear. Epidemiological studies suggest that the host inflammatory response may be an important factor in mediating different disease outcomes. Here we used a murine model to experimentally demonstrate that indeed the host inflammatory response is an important factor for CCHFV pathogenesis. Genetic disruption of a specific pathogen sensing pathway or an important inflammatory cytokine signaling pathway afforded the host a survival advantage. Additionally, pharmacological targeting of cytokine activity also conferred protection to the infected host. We also found that a strain of CCHFV that does not cause lethal disease in mice, also does not induce as potent of an inflammatory cytokine response as lethal strains, despite similar levels of replication. Our findings provide evidence that indeed the host response is an important component of the CCHFV pathogenic process, thus identifying host targets for pharmaceutical intervention.

## Introduction

Crimean-Congo hemorrhagic fever virus (CCHFV) is a tick-borne virus present throughout Africa, Asia and Europe [1–4]. In humans, CCHFV causes a febrile and lethal disease characterized by coagulopathy, liver injury and thrombocytopenia [5–9]. Human infection ensures following exposure to infected ticks or from exposure to infected animals during slaughter of livestock such as ostriches, cattle, and sheep [4,10]. Nosocomial infections also place hospital staff at significant risk [7,11]. The mortality rate of CCHF ranges from 3–30% and is suspected to depend on multiple factors including viral strain, speed of diagnosis, and access to emergency health care [3]. There are currently no licensed vaccines or therapeutics to prevent or treat CCHFV, although ribavirin may provide some therapeutic benefit [12]. CCHFV is endemic in a large geographical area and is emerging in to new regions, including Western Europe [6]. As a result CCHFV has been declared a WHO priority pathogen.

Type I interferon (IFN-I) activity has a substantial role in host susceptibility to severe CCHF. Human data suggests that people with TLR8/9 or TLR3 polymorphisms that limit IFN-I activation are more prone to developing severe disease [13,14]. Furthermore, rodents lacking IFN-I activity by genetic ablation of the IFN-I receptor (IFNAR-1), STAT1 or STAT2 activity are highly susceptible to lethal CCHFV infection, whereas wild-type animals with intact IFN-I activity do not develop disease [15–22]. In A549 cells, CCHFV triggers the mitochondrial antiviral signaling (MAVS) protein pathway through interactions with retinoic acid indictable gene (RIG-I) and melanoma differentiation-associated protein 5 (MDA5) [23]. This leads to suppression of viral replication through activation of antiviral systems, including ISG56, MxA, and IFN-β [23]. In addition to IFN-I activity, proinflammatory cytokines such as TNF-α and IL6, may also contribute to severe human disease caused by CCHFV [24–26].

How host sensing pathways and inflammatory cytokine activity determine disease outcome is unclear as the host response against CCHFV has been largely unexplored due to the limited number of animal systems available [27]. Previously, we adapted a transient IFN-I antibody blockade model to study CCHFV pathogenesis in transgenic mice [19,20,28]. For these studies IFN-I signaling was blocked using a murine non-cell depleting monoclonal antibody (mAb) targeting the IFNAR-1 subunit of the mouse IFN-α/β receptor (MAb-5A3) [19,20,28–31]. Similar to genetic knockout mice, IFN-I antibody blockaded mice develop a severe and fatal disease analogous to humans. As in humans, the primary target organ in mice is the liver. Concomitant with liver damage is the activation of inflammatory cytokines including TNF-α, IL-6 and IL-1 [19]. Here, we used this murine system to explore the role that MAVS and inflammatory cytokines play in CCHFV pathogenesis. Our findings reveal that MAVS-driven IFN-I signaling did not increase murine permissiveness to lethal disease. However, in contrast to control mice, animals lacking MAVS were completely protected from lethal disease when IFN-I signaling was blocked and exhibited a more limited inflammatory response. Subsequent analysis revealed that a specific host cytokine pathway was important for progression of lethal disease. These findings implicate the host response as an important contributor to CCHFV pathogenesis.

## Results

### MAVS-dependent signaling is required for CCHFV-mediated acute disease in IFN-I blockaded mice

**Fig 1:**
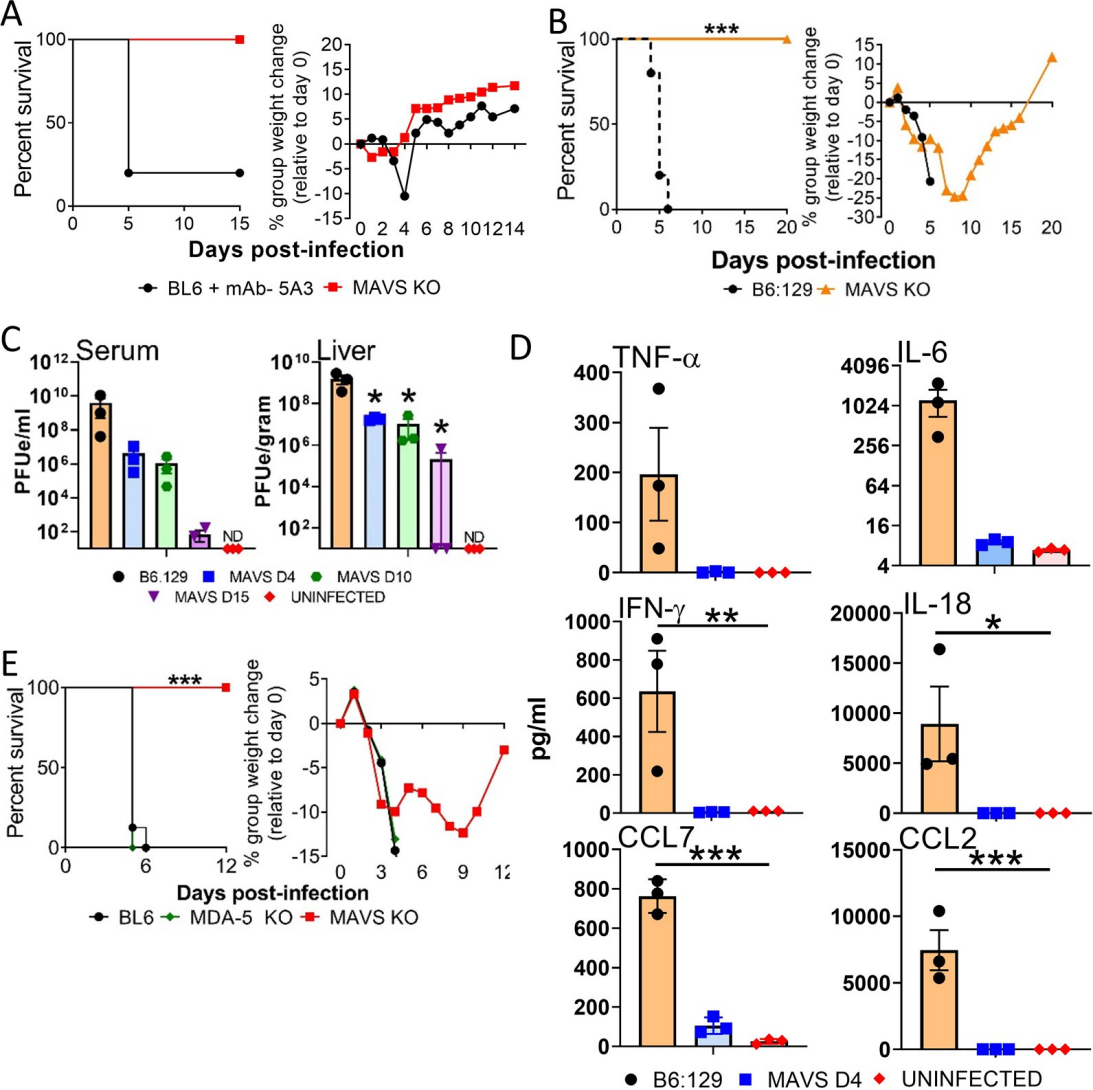
MAVS KO mice with IFN-I signaling blocked are protected against lethal infection by CCHFV. **A.** C57BL/6 mice or MAVS KO mice (n = 5/group) were infected with CCHFV and survival and weight loss were monitored and plotted using Prism software. Only WT mice were treated with MAb-5A3 24h after infection to block IFN-I. IFN-I was not blocked by antibody in the MAVS KO mice. **B**. B6.129 or MAVS KO mice (n = 6/group) were infected with CCHFV and both groups treated 24 h post-infection were treated with mAb-5A3. Survival and weight loss were monitored for 20 days and plotted using Prism software. Survival significance was determined by log-rank analysis; ***p<0.0001. **C.** Viral RNA in serum and liver was examined on day 4, 10 and 15 by RT-qPCR (n = 3 per group). Mean titers +/- SEM of the estimated PFUs (PFUe) were graphed. The dashed black line denotes limit of detection. Statistical significance was determined by one-way ANOVA; *p<0.05. **D.** Monocyte chemoattractants and inflammatory cytokines were measured from the serum (n = 3 per group) of CCHFV infected mice on day 4 using a multiplex system. Statistical significance compared to uninfected controls was determined by one-way ANOVA; *p<0.05, ***p<0.001. **E.** C57BL/6, MDA5 KO or MAVS KO mice (n = 8 per group) were infected with CCHFV and 24 h post-infection were treated with MAb-5A3. Survival and weight loss were monitored for 20 days. Significance determined by log-rank analysis (p<0.0001).

Because MAVS is important in the IFN-I response, we initially investigated if it alone played an important role in host susceptibility to CCHFV infection. Mice lacking MAVS (MAVS KO), but with otherwise intact IFN-I signaling, were not susceptible to CCHFV infection by the murine lethal strain Afg09-2990 and exhibited no weight loss or mortality. This was in contrast to the extensive weight loss and 80% mortality in CCHFV strain Afg09-2990 infected non-transgenic wild-type (WT) mice (C57BL/6) treated with a monoclonal antibody (mAb)-5A3 to block IFN-I signaling (**Fig 1A**). This finding indicated that loss of MAVS does not enhance susceptibility of mice to CCHFV. Because MAVS induces inflammatory signaling pathways independent of IFN-I activation [32,33], we next evaluated CCHFV infection in MAVS-deficient mice in the presence of an antibody-induced IFN-I signaling blockade. IFN-I antibody blocked and infected MAVS KO mice lost weight starting on day 2 and continued decreasing until day 9 before recovering. (**Figs 1B and S1**). Weight loss was similar to IFN-I blocked and infected WT mice (B6:129). Despite weight loss, all MAVS KO mice survived infection, whereas all WT mice succumbed to disease by day 6. CCHFV infection in IFN-I blockaded mice was repeated in 19 MAVS KO mice over multiple studies with universal survival, contrasting against 100% mortality in control WT mice (Log-rank; p<0.0001) (**Figs 1B and 1E and S1**).

Levels of viral genome in the liver and serum on day 4 post-infection were reduced in mAb-5A3 treated MAVS KO mice compared to WT mice treated with mAb-5A3 (**Fig 1C**). The difference in viral load was statistically significant in the liver, but not serum (one-way ANOVA; p<0.05). Viral RNA was also detected on day 10, and to a lesser extent on day 15 in both the liver and serum of MAVS KO animals. MAVS deficient mice had a large reduction in the cytokines TNF-α, IL-6, IFN-γ, IL-18 and the chemokines CCL7 and CCL2 on day 4 compared to the infected WT mice (**Fig 1D**). MAVS signaling is transduced via MDA5 and RIG-I. However IFN-I blocked, CCHFV-infected MDA5 deficient animals did not have a survival advantage over control mice (**Fig 1E**). These findings indicated that loss of MAVS, but not MDA5, confers a significant survival advantage to CCHFV infected mice in the absence of IFN-I signaling.

**Fig 2:**
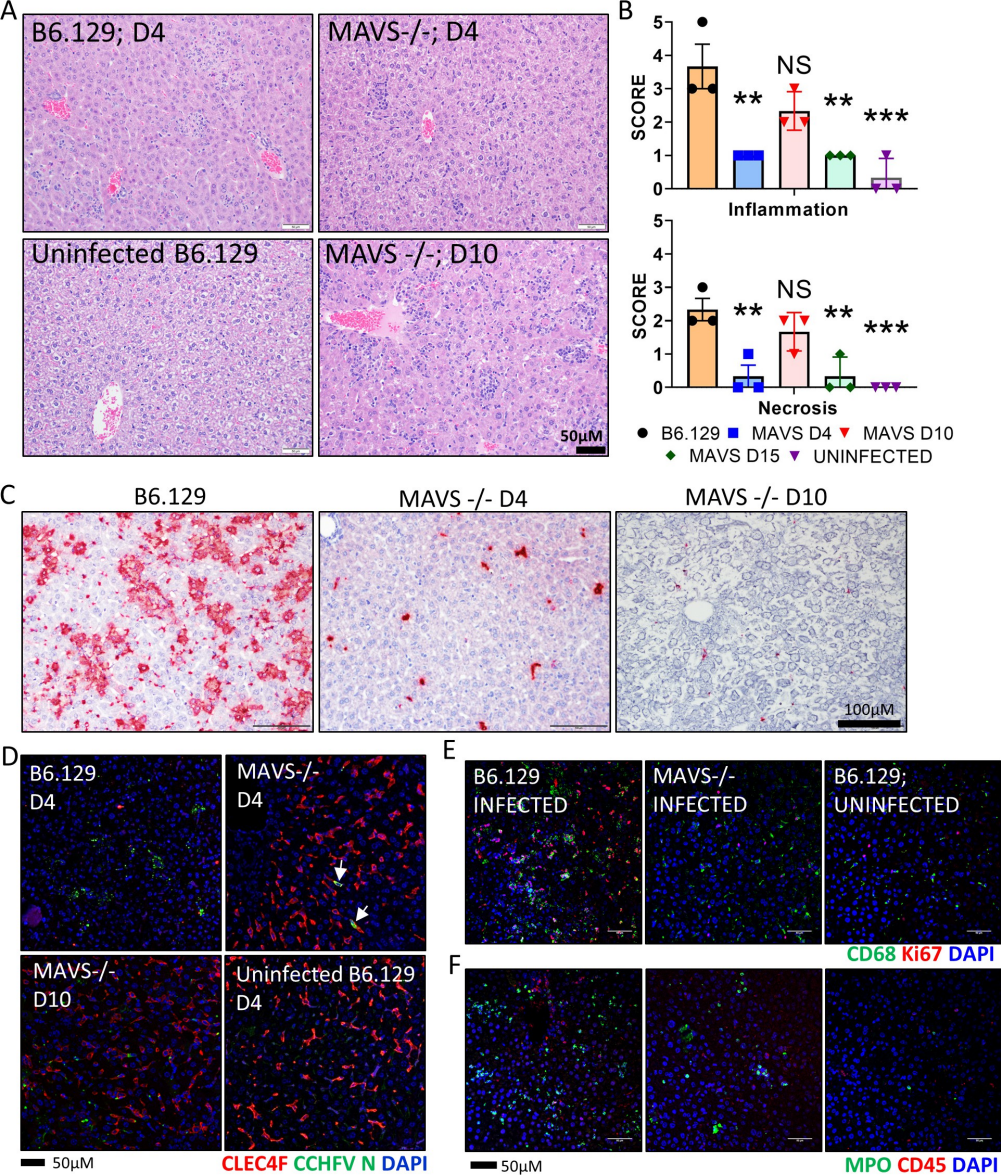
Hepatic injury is reduced in CCHFV infected MAVS KO mice. **A.** Representative H&E staining of livers from WT (Day 4) and MAVS KO mice infected with CCHFV in the presence of IFN-I blockade harvested on Day 4 and 10 (n = 3/group/time point). mAb-5A3 treated Day 4 B6.129 mice show increased liver pathology compared to MAVS KO mice or uninfected mice. Inflammation and necrosis were increased in the liver on day 10 in the MAVS KO mice, compared to the day 4 in the MAVS KO mice, but pathology was still less severe than in the WT (B6.129) mice. **B.** Pathology scores for WT (Day 4) or MAVS KO mice (Day 4, 10 and 15) were plotted for the indicated lesions (n = 3 per group). Statistical significance of infection in MAVS KO mice compared to infected controls was determined by one-way ANOVA; **p<0.05, ***p<0.001 or NS; not significant. **C** Representative ISH staining of livers. ISH stained tissue was counterstained with hematoxylin. **D.** Liver sections from infected (WT [B6.129] or MAVS KO) or uninfected WT (B6.129) mice were stained with anti-CLEC4F (red) and anti-CCHFV N protein antibodies (green). Cell nuclei were stained with DAPI. **E and F**. IFA demonstrates increased number of CD68^+^ macrophages (E, green) and Ki67^+^ proliferating cells (E, red) or MPO^+^ neutrophil granulocytes (F, green) and CD45^+^ leukocytes (F, red) in livers of WT (B6.129) mice compared to MAVS KO or uninfected mice. Nuclei are stained with DAPI (blue).

The liver is a key target organ of CCHFV in humans and in mice [3,9,19,34]. Histopathological changes in livers from CCHFV infected WT (B6.129) and MAVS KO mice in the presence of IFN-I signaling blockade were evaluated on day 4 for both strains of mice and day 10 and 15 for MAVS KO mice. On Day 4, WT mice developed hepatic lesions, marked by extensive inflammation with hepatocellular degeneration and necrosis (**Fig 2**). Kupffer cell hypertrophy was evident in the sinusoids, and occasional periportal oval cell hyperplasia. In contrast, liver pathology was marginal on day 4 in MAVS KO mice and was characterized by minimal multifocal inflammation. Liver pathology was more prevalent in MAVS KO mice on day 10, with increased inflammation and moderate hepatocelluar necrosis. However, these hepatic lesions were relatively mild in comparison to the damage observed in infected, WT mice on day 4. By day 15, liver injury is predominantly absent in MAVS KO animals, with only minimal inflammation detected (**Fig 2B**).

In situ hybridization (ISH) showed viral genomic RNA was prevalent in WT mouse livers on day 4, but the level of staining was comparatively reduced in MAVS KO mice on day 4 and day 10 (**Fig 2C)**. The liver ISH staining pattern suggested that Kupffer cells were the primary cells targeted by CCHFV in the MAVS KO mice on day 4. We previously reported that on day 4 infected WT mice lacking IFN-I activity have nearly a complete loss of Kupffer cells indicated by an absence of CLEC4F^+^ staining [19]. Here, WT mice also had an extensive loss of CLEC4F^+^ staining on day 4 (**Fig 2D**). In contrast, Kupffer cell loss was absent in infected MAVS KO mice with levels of CLEC4F^+^ staining similar to uninfected mice. In MAVS KO mice, viral nucleocapsid (N) protein was predominantly localized to Kupffer cells on day 4. N protein staining of MAVS KO mice became more disseminated on day 10, present in CLEC4F positive and negative cells. At the day 4 time point, an influx of CD68^+^ monocytes/macrophages and Ki67+ proliferating cells and an increase in myleoperoxidase (MPO)^+^ neutrophil granulocytes and CD45^+^ leukocytes in the WT animals was detected (**Fig 2E and 2F**). Staining of MPO^+^ neutrophil granulocytes was reduced in MAVS mice on day 4, but higher than uninfected mice. Staining of Ki67^+^ proliferating cells and CD45^+^ leukocytes in MAVS KO mice was similar to uninfected animals. However, CD68^+^ staining was still present in MAVS KO mice, but the staining pattern was more indicative of Kupffer cells which are CD68^+^. These results demonstrated that the loss of MAVS ameliorates CCHFV-induced hepatic inflammation.

**Fig 3:**
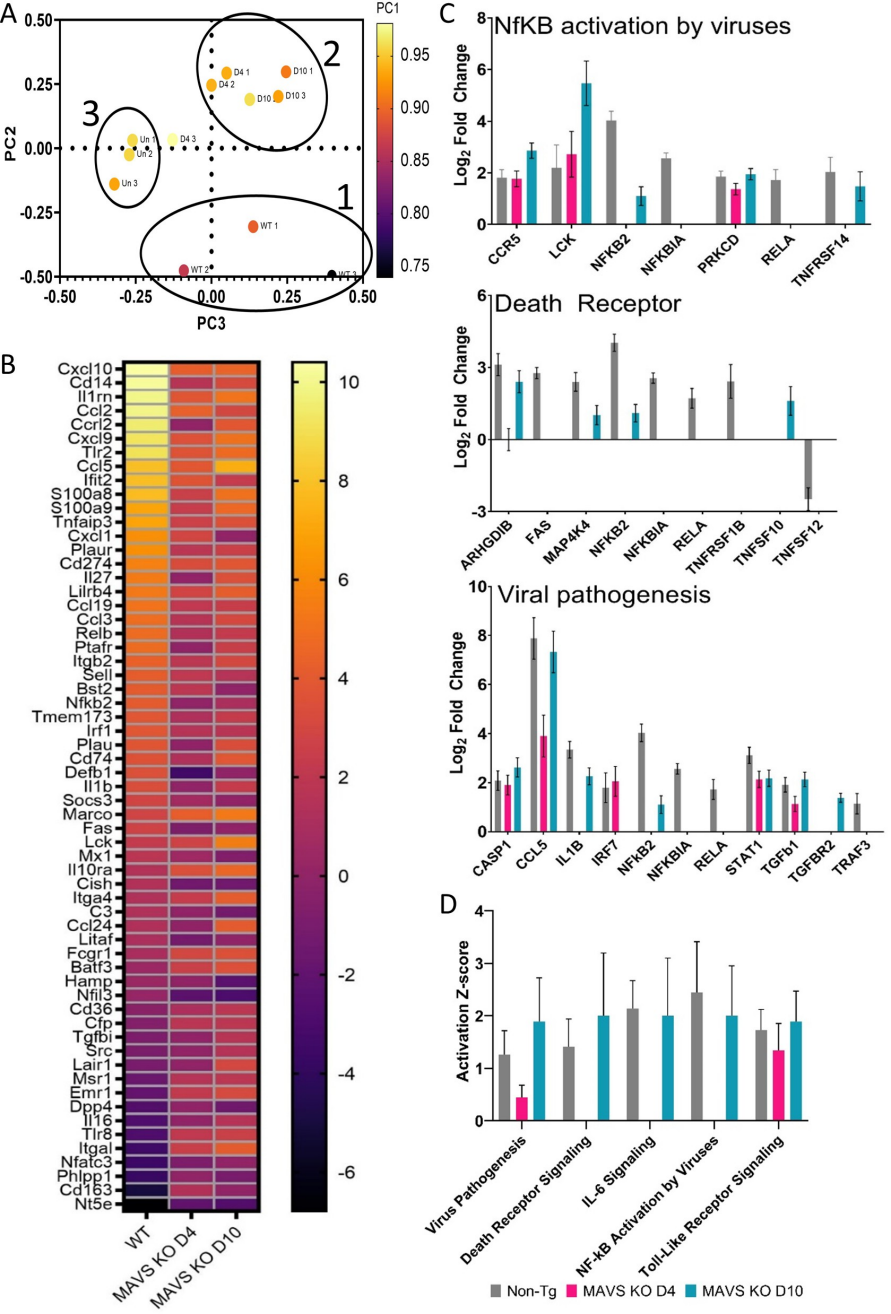
Transcriptional activation in CCHFV infected livers compared to mock infected mice. **A.** Transcriptional activation in liver homogenates from mice (n = 3 per group/time point) infected with CCHFV with IFN-I blockade or mock infected were examined by NanoString. Principal Component Analysis (PCA) was performed by nSolver analysis software. Circles designated as infected WT mice (1), infected MAVS KO mice day 4 and 10 (2) and uninfected mice (3). The uncircled data point is from an unclustered MAVS KO infected mouse from D4. **B.** Heat map showing individual changes in indicated CCHFV infected mice relative to uninfected control mice. **C**. Log2 fold changes for selected genes involved in the indicated pathways were determined along with statistical significance. **D.** Activation score (Z-score) for the indicated pathways in infected WT or MAVS KO mice versus uninfected mice. Activation value was determined by Ingenuity Pathway Analysis software.

Gene expression profiling of 297 genes associated with host immunology and inflammatory processes was examined using the NanoString nCounter platform and total RNA isolated from mouse liver homogenates (n = 3/time points) on day 4 (WT, MAVS KO and uninfected WT mice) and day 10 (MAVS KO). The three groups of animals (infected WT, MAVS KO and uninfected WT mice, all IFN-I blocked) had bulk transcript profiles that were uniquely distributed by principal component (PC) analysis **(Fig 3A)**. MAVS KO day 4 and day 10 groups mostly clustered together with one exception, but uninfected and infected WT mice clustered separately. Transcriptomic profiling demonstrated that on day 4 MAVS KO infected mice have blunted inflammatory responses compared to WT, infected mice. On day 10, some inflammatory response gene signatures are restored to levels similar to those of infected WT mice (**Fig 3B**). In day 4 samples, pathway scoring using Ingenuity Pathway Analysis (IPA) from QIAGEN indicated decreases in gene signatures for viral pathogenesis, death receptor signaling, IL-6 signaling, and NF-kB activation by viruses in MAVS KO livers (**Fig 3C and 3D**). In the MAVS KO group, several pathways were not elevated above non-infected animals, including IL-6, HMGB1, and iNOS; however, by day 10 Z-scores for those pathways were similar to infected WT mice on day 4 **(S2 Fig)**. These findings indicated that MAVS deficiency delays inflammatory responses in CCHFV infected mice compared to WT animals.

### CCHFV lethality is limited in mice lacking intact TNF-α receptor signaling

**Fig 4:**
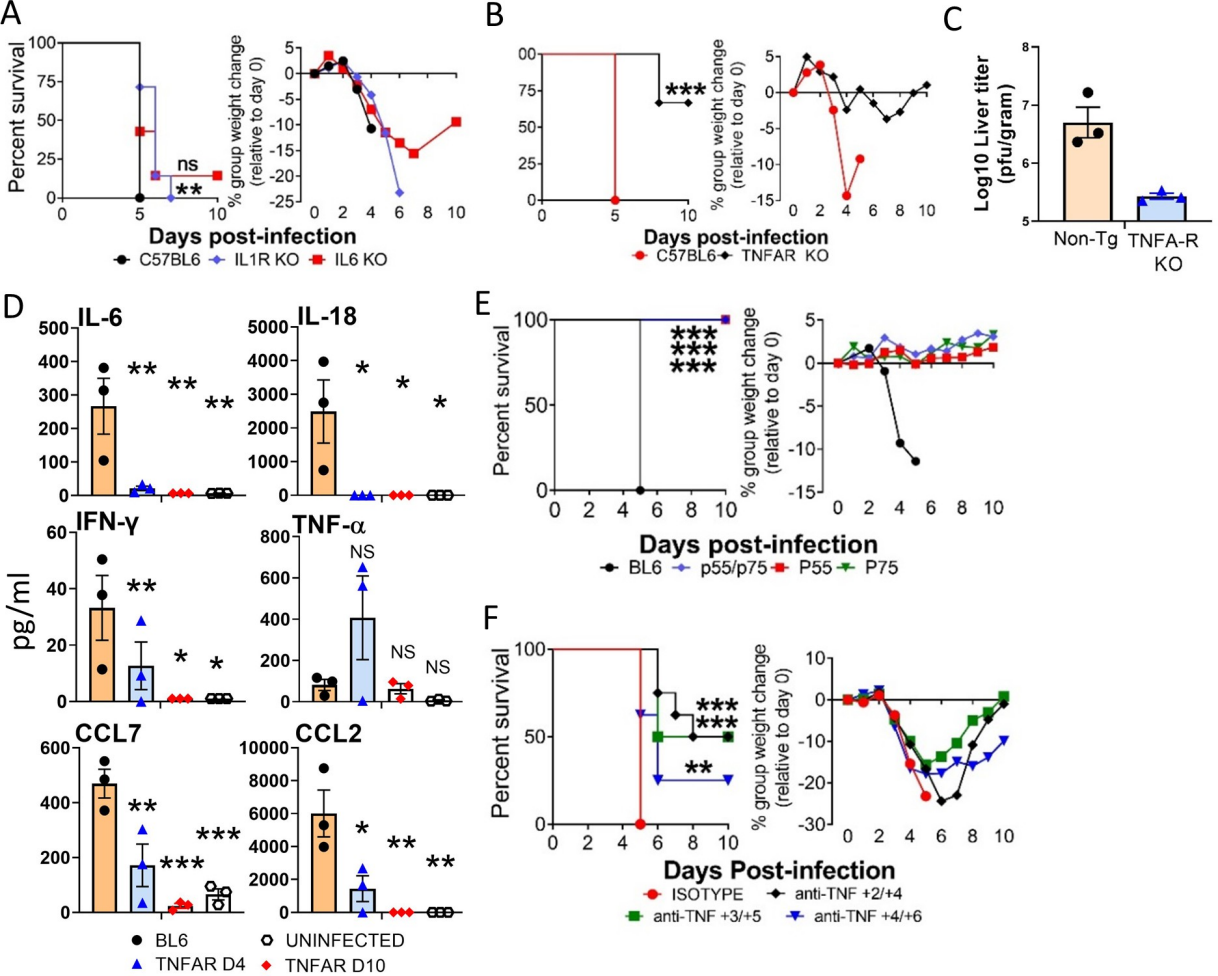
Mice lacking TNFA-R signaling are partially protected against CCHFV. **A.** WT (C57BL/6), IL-1R KO and IL-6 KO mice (n = 5 per group) were infected with CCHFV and survival and weight loss were monitored as above. Mice were treated with MAb-5A3 24h after infection to block IFN-I. Significance in time to death was determined by log-rank analysis; **p<0.05. NS: not significant. **B**. WT (C57BL/6) or TNFA-R DBL KO mice (n = 6 per group) mice were infected with CCHFV and injected with MAb-5A3 a day after challenge. Weights and survival were monitored for 10 days post-infection. Significance in survival and the delay in time to death was determined by log-rank analysis; ***p<0.0001. **C.** Viral load in day 4 livers was quantified by plaque assay (n = 3 per group). Dashed line shows the limit of detection. **D**. Monocyte chemoattractants and inflammatory cytokines were measured from the serum of uninfected mice or CCHFV infected WT (BL6) or TNFA-R DBL KO mice (n = 3 per group) mice on day 4 (BL6 and TNFA-R DBL KO mice) and 10 (TNFA-R DBL KO mice) using a multiplex system. Statistical significance compared to infected controls was determined by one-way ANOVA; *p<0.05, **p<0.01, ***p<0.001. **E.** C57BL/6, TNFA-R DBL KO or mice lacking either TNF receptor (p55 or p75) mice (n = 8 per group) were infected with CCHFV and injected with MAb-5A3 as above. Weights and survival were monitored for 10 days post-infection. Survival significance versus C57BL/6 wild-type mice was determined by log-rank analysis; ***p<0.0001. **F.** C57BL/6 (n = 8 per group) were infected with CCHFV and IFN-I antibody blockade was established 24 h post-infection as above. Mice were treated with anti-TNF-α neutralizing antibody or isotype control as indicated. Weights and survival were monitored for 10 days post-infection. Significance of delay in time to death and overall increase in survival compared to isotype control animals was determined by log-rank analysis; **p<0.05, ***p<0.0001.

Because IFN-I blocked and infected MAVS-deficient animals had limited cytokine activity, we next investigated if apex cytokines IL-1, IL-6, and TNF-α were critical for CCHFV pathogenesis. Mice deficient in IL1-receptor (IL-1R), IL-6 or both TNF-α receptors (TNFA-R DBL KO) or WT mice were infected with the murine lethal CCHFV strain Afg09-2990 and IFN-I was blocked at 24 h post-exposure. Survival and weight loss monitored for 10 days. IL1R KO mice had a modest, but significant (log-rank; p<0.05) increase in time to death over WT control mice (**Fig 4A**). IL-1R KO mice also had a slight delay in weight loss compared to WT animals, but all animals succumbed to disease similar to controls. IL-6 KO mice had no survival advantage over WT animals and only a single mouse survived infection (**Fig 4A**). In contrast, a much larger survival advantage was observed in TNFA-R DBL KO mice and most survived challenge or had a significant delay in mean time to death (**Fig 4B**). Weight loss was also reduced in these mice compared to control animals. Viral load was slightly reduced in the TNFA-R DBL KO mice compared to the WT animals on day 4, but this difference was not significant (**Fig 4C)**. Additionally, strain Afg09-2990 infected TNFA-R DBL KO mice had lower levels of serum inflammatory cytokines and chemokines on day 4 and 10 compared to WT animals on day 4 (**Fig 4D**).

There are two TNFA receptors TNFA-R1 (p55) and TNFA-R2 (p75). To establish which receptor pathway was most critical to CCHFV-indicated liver injury, mice lacking the individual receptor or lacking both receptors and WT mice were infected with CCHFV Afg09-2990 and IFN-I antibody blocked 24 h post-infection (**Fig 4E)**. All control mice died by day 5 after a period of weight loss, however, neither the individual nor double receptor KO mice succumbed to disease or showed weight loss. Similar to the receptor KO mice, TNF-α neutralizing antibody also protected against CCHFV infection in a post-exposure setting (**Fig 4F**). We found that 4/8 mice survived challenge when treatment began on day +2/+4 and +3/+5 and non-surviving animals showed a delayed time to death. This delay in mean time to death and increase in survival compared to isotype treated animals was significant. Protection was more limited when treatment was given on day +4/+6. Together, these data indicated that loss of TNFA-R signaling or targeting of TNF-α by neutralizing antibody is protective against CCHFV.

**Fig 5:**
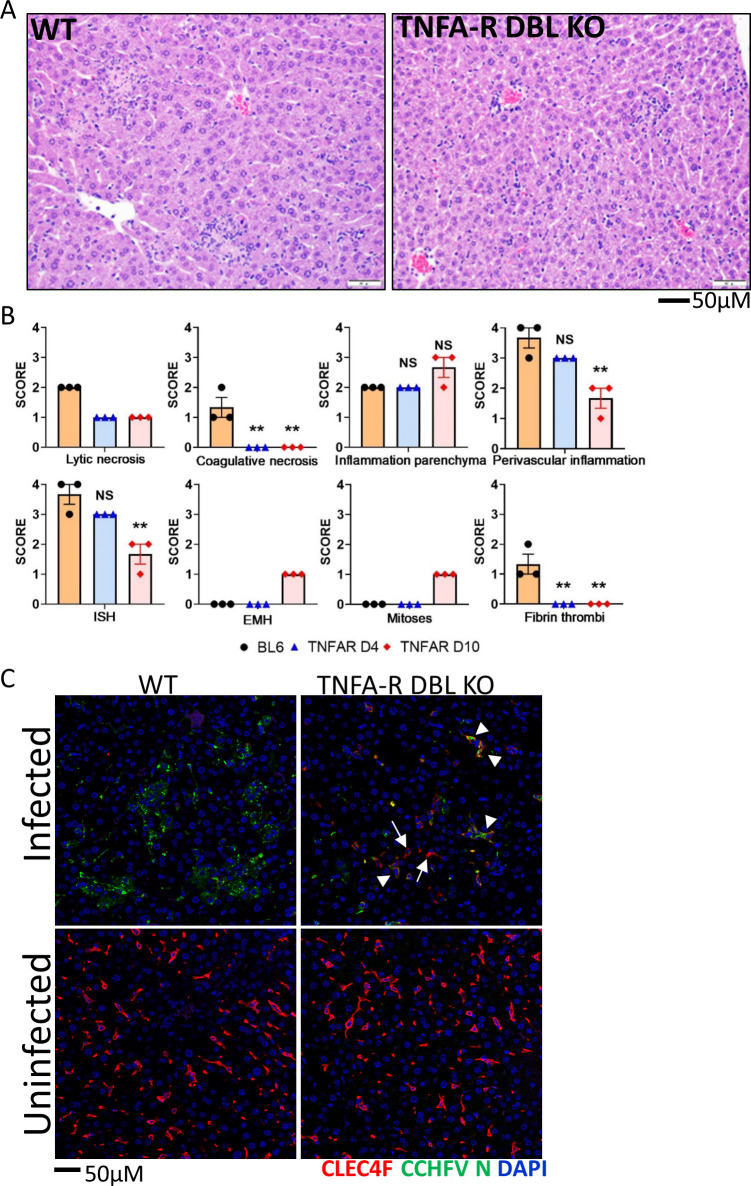
Liver injury is reduced in CCHFV infected mice missing TNFA-R signaling. **A** Representative H&E staining of livers from WT (C57BL/6) and TNFA-R DBL KO mice infected with CCHFV in the presence or absence of IFN-I blockade harvested on Day 4. Livers have multifocal areas of random, lytic and single cell hepatocyte necrosis with accompanying inflammation which are larger in WT mice compared to the smaller, more discrete areas of necrosis in TNFA-R DBL KO mice. **B.** Pathology scores were plotted for the indicated lesions or ISH staining (n = 3/group/time point). Statistical significance of day 4 and day 10 infected, TNFA-R DBL KO mice compared to infected controls was determined by one-way ANOVA; **p<0.05 or NS; not significant. **C**. Liver sections from day 4 infected or uninfected WT (C57BL/6) or TNFA-R DBL KO mice were stained with anti-CLEC4F (red) and anti-CCHFV N protein (green) antibodies. Cell nuclei were stained with DAPI. Arrows point to CLEC4F+ cells and arrow heads point to infected CLEC4F+ cells.

**Fig 6:**
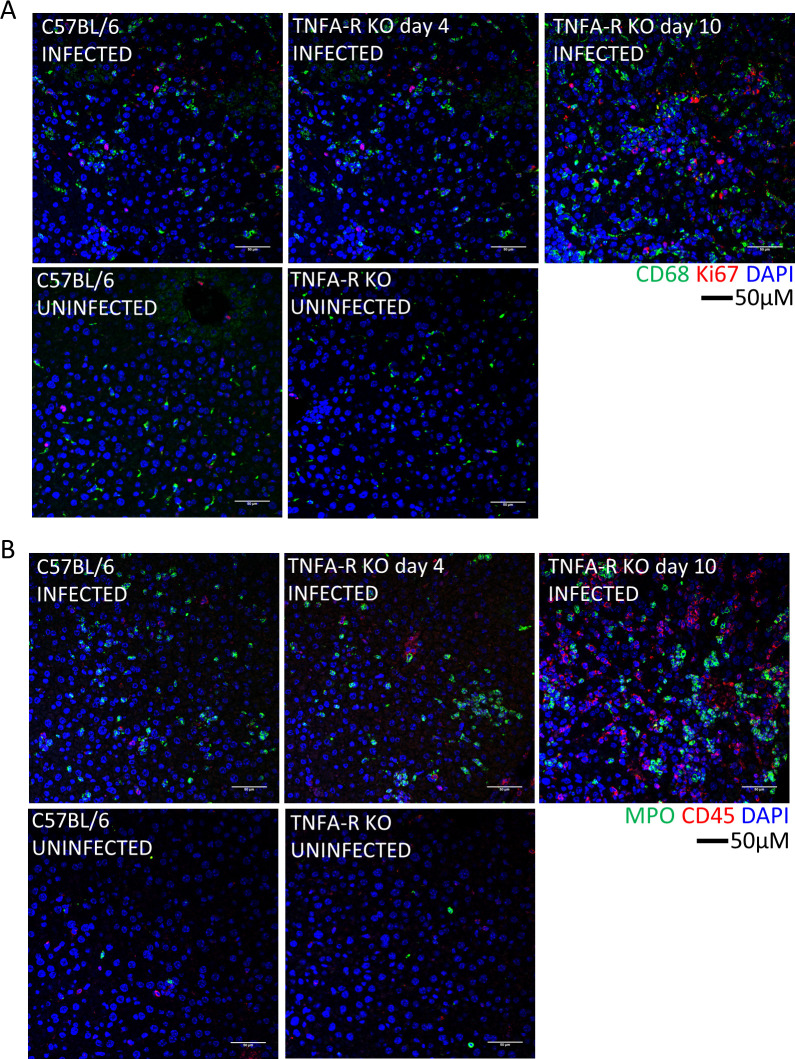
Immune cells in the livers of WT and TNFA-R KO mice. Representative IFA demonstrates presence of CD68^+^ macrophages (A, green) and Ki67^+^ proliferating cells (A, red) or MPO (B, green), and CD45^+^ leukocytes (B, red) in livers of infected animals compared to uninfected at the indicated time points. Nuclei are stained with DAPI (blue).

Histopathologically, TNFA-R DBL KO mice had a lesser degree of lytic necrosis on both days 4 and 10, compared to WT mice on day 4 **(Fig 5A and 5B**). However, animals had similar levels of parenchymal inflammation, which increased in TNFA-R KO mice on day 10. Additionally, in marked contrast to WT animals, no fibrin thrombi or coagulative necrosis was observed in mice lacking TNFA-R signaling at any time point. Furthermore on day 10, we detected the presence of mitotic figures and extramedullary hematopoiesis (EMH) in the livers of TNFAR DBL KO mice (**Figs 5B and S3A**). Liver injury in TNFA-R DBL KO mice was accompanied by a decrease in Kupffer cells on day 4, but this loss was not as severe as the near complete lack of detectable CLEC4F^+^ Kupffer cells in infected, WT mice (**Fig 5C**). Also at this time point, viral antigen was detectable in both CLEC4F^+^ Kupffer cells and in non-CLEC4F cells. On day 10, the Kupffer cell population rebounded to levels of uninfected controls (**S3B Fig**). Viral antigen was observed both within Kupffer cells and in other cells within the liver. We also saw increases in CD68^+^ and Ki67^+^ cells in TNFAR DBL KO mice on day 4 that increased on day 10 (**Fig 6A**). We also noted an influx of MPO^+^ and CD45^+^ cells in the livers of TNFA-R DBL KO that was similar to WT mice on day 4 and increased MPO signatures were seen on day 10 (**Fig 6B**). These data showed that while loss of TNFA-R signaling did not block inflammatory cells infiltration in response to CCHFV infection, liver injury was slightly more attenuated compared in WT animals. Additionally, livers in TNFA-R BDL KO mice showed signs of liver recovery at later time points, including EHM and mitotic figures.

### CCHFV strain Kosova Hoti is attenuated in mice and produces blunted inflammatory responses

**Fig 7:**
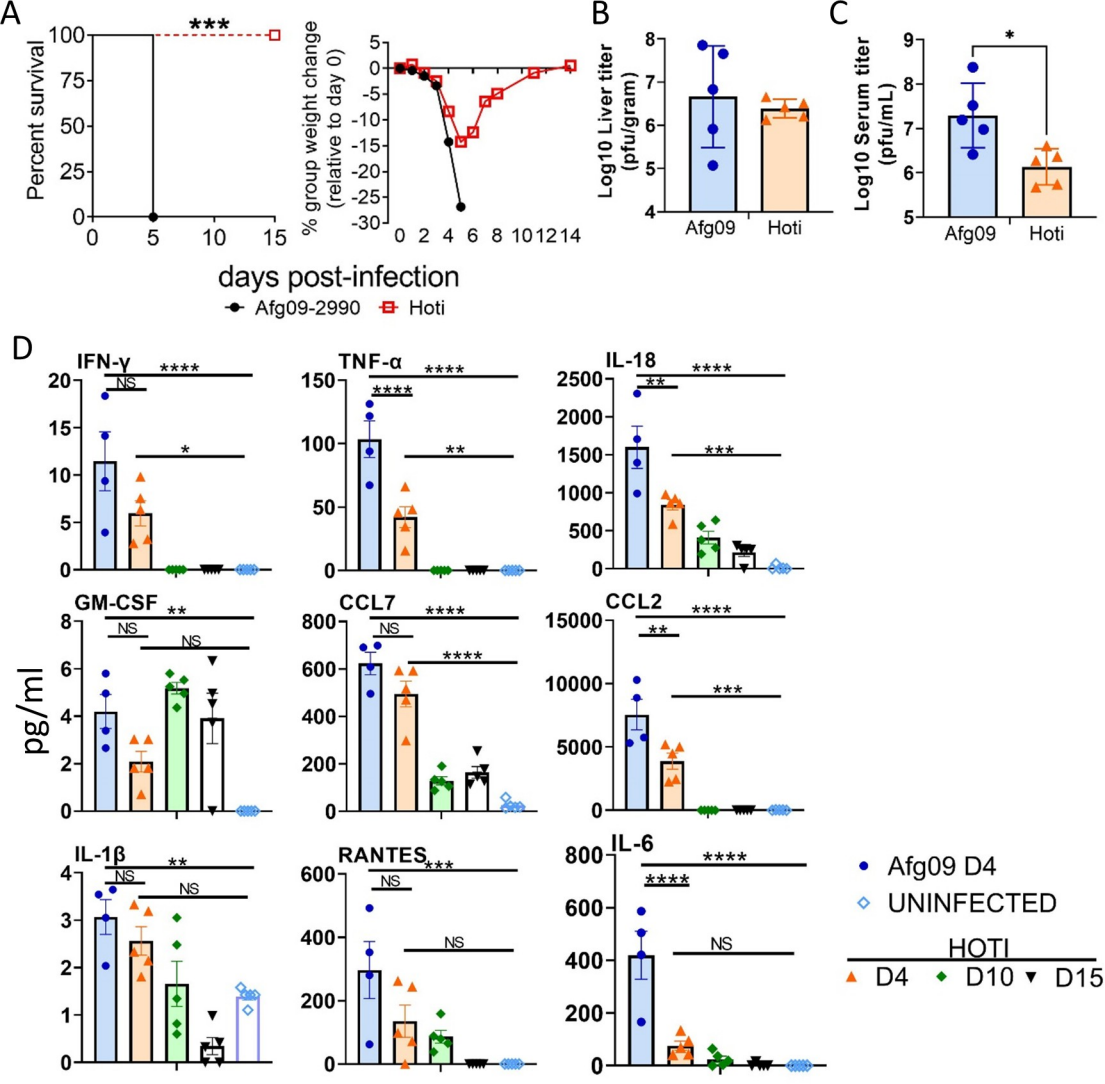
Infection of mice with CCHFV strains Afg09-2990 and Hoti. **A.** C57BL/6 mice (n = 8 per group) mice were infected with CCHFV strains Afg09-2990 or strain Hoti and injected with MAb-5A3 a day after challenge. Weights and survival were monitored for 15 days post-infection. Significance determined by log-rank analysis; ***p<0.0001. **B.** Liver viral titers were determined on day 4 (n = 5 mice/group) by plaque assay on cell monolayers and plotted as PFU/gram of tissue. **C**. Viremia on day 4 was determined (n = 5 per group) as in panel B and plotted as PFU/ml. **D.** Monocyte chemoattractants and inflammatory cytokines were measured from the serum of CCHFV strain Afg09-2990 (day 4), strain Hoti (day 4, 10 and 15) infected mice (n = 5 per group) or uninfected mice using a multiplex system. Statistical significance compared to uninfected controls was determined by one-way ANOVA; *p<0.05, **p<0.01, ***p<0.001.

CCHFV strain Kosova Hoti (Hoti) is less virulent in IFN-I deficient mice [35] compared to the murine lethal strain Afg09-2990. We compared the inflammatory response of the non-lethal strain against the lethal strain Afg09-2990 in antibody-mediated IFN-I blockaded mice. C57BL/6 mice were infected with CCHFV strain Afg09-2990 or strain Hoti and IFN-I was blocked as above. Weights and survival were monitored for 15 days (**Fig 7A**). On day 2, Afg09-2990 infected mice began to lose weight and all mice succumbed to disease by day 5. Strain Hoti infected mice also began to lose weight on day 2 through day 5, but all animals survived infection. The difference in survival was significant compared to Afg09-2990 (log-rank; p<0.0001). Liver viral load, determined by plaque assay, was not statistically different between these two strains on day 4 (**Fig 7B**). However, viremia was significantly higher for strain Afg09-2990 compared to strain Hoti (**Fig 7C**). Cytokine and monocyte chemokine activity were compared between mice infected with strain Afg09-2990 and Hoti on days 4, and also for strain Hoti on days 10 and 15. TNF-α, IL-6, IL-18, IL-1β, IFN-γ and GM-CSF activity were higher in Afg09-2990 infected mice compared to strain Hoti on day 4, with several differences reaching statistical significance (two-way ANVOA; p<0.05) (**Fig 7D**). Similarly, CCL2, CCL4, CXCL1, and CXCL10 were higher in Afg09-2990 infected mice compared to strain Hoti. On days 10 and 15, the levels of cytokines and chemokines in Hoti infected animals generally diminished, with the exception of GM-CSF, which increased on day 10.

Additionally, IFN-I blocked, Rag2-deficient mice succumbed to disease by day 6 when infected with strain Afg09-2990 mice, whereas Hoti infected animals did not meet euthanasia criteria until day 20 (**S4A Fig**). This delay in time to death was significant (log-rank; p<0.05). In these mice, strain Hoti infection produced lower TNF-α responses compared to Afg09-2990 on day 4 post-infection as determined by ELISA (**S4B Fig**). Liver enzymes, however were similar in both groups on day 4 (**S4C Fig**). Overall, these data indicated that strain Hoti does not cause a lethal infection in immune intact, IFN-I blockaded mice, and lethality in mice deficient in adaptive immunity was significantly delayed. Moreover, strain Hoti infected animals have blunted inflammatory cytokine and chemokine responses compared to the lethal strain Afg09-2990.

**Fig 8:**
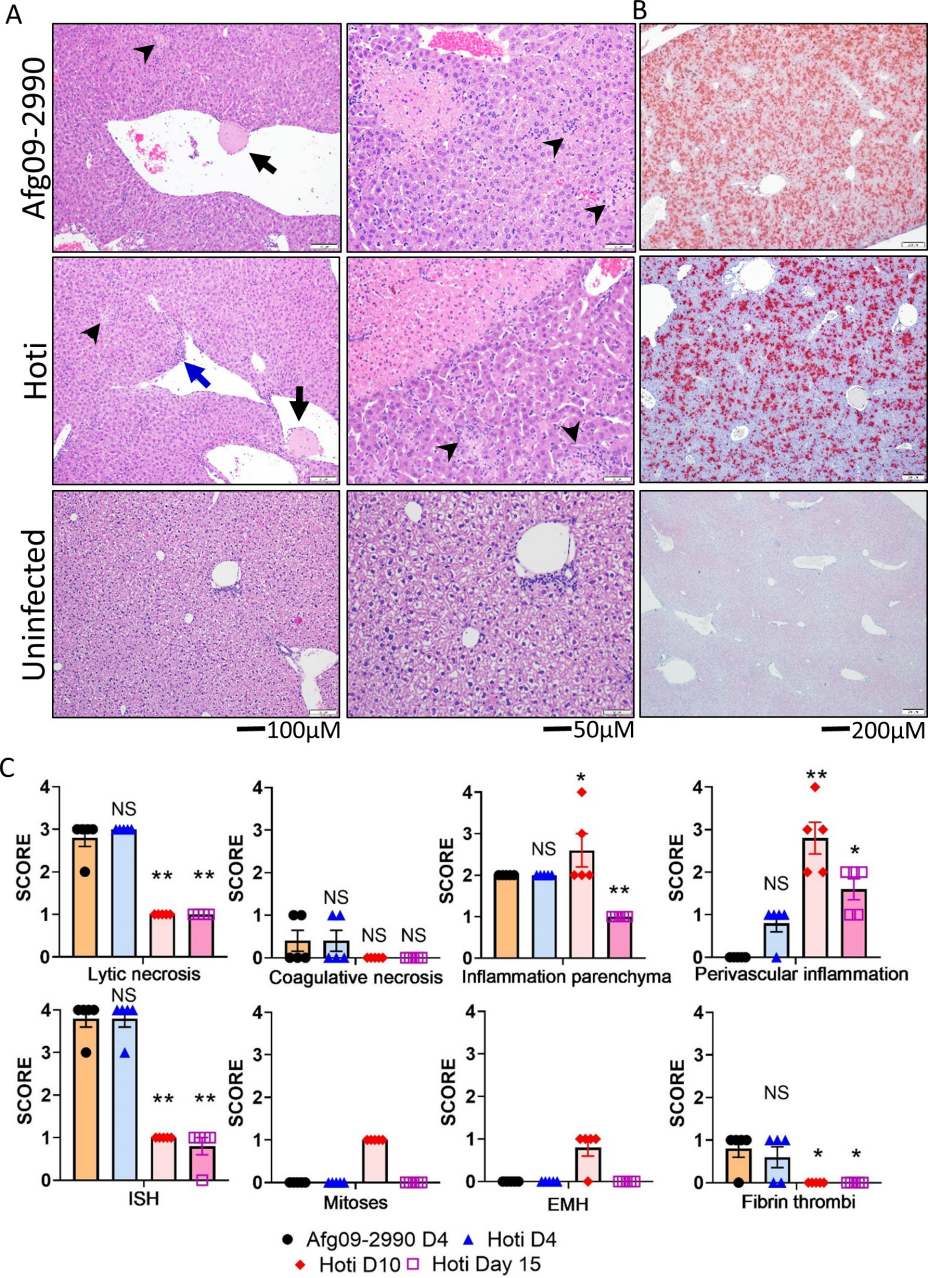
Liver lesions produced by CCHFV strain Afg09-2990 and strain Hoti. Representative H&E (**A**) and ISH (**B**) staining of livers Afg09-2990 and Hoti infected mice or uninfected animals as indicated. Both Afg09-2990 and Hoti infected livers have fibrin thrombus in a central vein (black arrows) as well as multifocal areas of necrosis (arrowheads). In Hoti infected, but not as much in Afg09-2990, we noted liver mononuclear inflammation adjacent to the portal vein (blue arrow). 20X panels show of coagulative necrosis at upper left with smaller foci of lytic necrosis and inflammation (arrowheads). **C.** Pathology scores were plotted for the indicated lesions or ISH staining (n = 5 per group). Statistical significance of day 4 and day 10 infected, TNFA-R DBL KO mice compared to infected controls was determined by one-way ANOVA; *p<0.05, **p<0.01. NS; not significant.

Liver pathology between strain Hoti and Afg09-2990 was compared on day 4 (n = 5 per group). Liver lesions were comparable between both strains, with similar levels of lytic necrosis, coagulative necrosis, thrombi formation and parenchymal (specifically affecting hepatocytes) inflammation (**Fig 8A and 8C**). There were higher levels of inflammation around central veins and in portal areas (termed “perivascular”) in Hoti infected mice. Because the mice survive, livers (n = 5 per time point) from Hoti infected mice were also examined on day 10 and 15. On day 10, livers from Hoti infected animals had a decrease in lytic necrosis with only low numbers of individual apoptotic or necrotic hepatocytes present (i.e. single cell necrosis), a slight increase in parenchymal inflammation and a larger increase in perivascular inflammation (**S5 Fig**). On day 15, low numbers of individual apoptotic or necrotic hepatocytes remained, but parenchymal and perivascular inflammation both decreased. Coagulative necrosis and fibrin thrombi were absent on both day 10 and 15. Also on day 10 was the presence of extra medullary hematopoiesis (EMH) in most animals (4/5) and mitotic figures within hepatocytes in all mice (5/5). Both EMH and mitotic figures were absent in day 15 animals. High levels of viral genomic RNA were detected in both Afg09-2990 and Hoti infected livers by ISH on day 4 post-exposure (**Fig 8B and 8C**). RNA levels for Afg09-2990 and Hoti were present at similar levels. These viral RNA ISH levels decreased in Hoti livers on day 10 and were undetectable in one animal on day 15 (**Fig 8C**).

**Fig 9:**
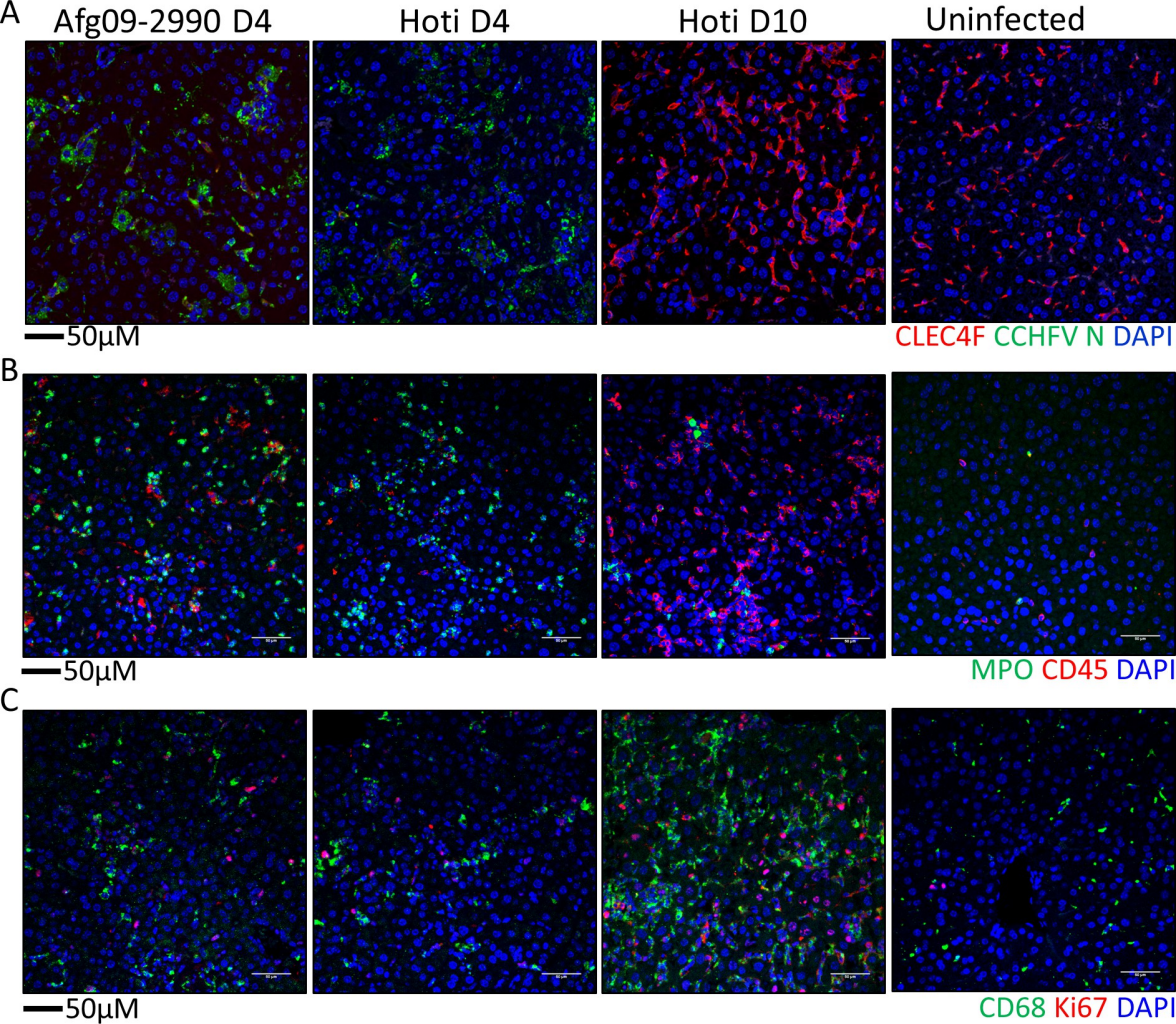
Immune cell levels in Hoti and Afg09-2990 infected mice. **A.** Liver sections from day 4 Afg09-2990 or Hoti infected or uninfected mice were stained with anti-CLEC4F (red) and anti-CCHFV N protein (green) antibodies. **B.** Liver sections from were stained with MPO (green) and CD45 (red). **C.** IFA demonstrating presence of CD68^+^ macrophages (green) and Ki67^+^ proliferating cells (red) in livers of day 4 animals. For all panels, cell nuclei were stained with DAPI.

We also observed similar decreases in CLEC4F^+^ Kupffer cells in strain Afg09-2990 and Hoti infected mice coinciding with the presence of nucleoprotein (N) throughout the liver. In Strain hoti infected mice, CLEC4F^+^ Kupffer cells were restored to levels similar to uninfected mice by day 10 (**Fig 9A**). Furthermore, there were similar levels of infiltrating MPO^+^ granulocytes and CD45^+^ cells in the livers of Afg09-2990 and Hoti animals on day 4 (**Fig 9B**). Granulocytes were also elevated over control mice on day 10 in Hoti infected mice. Marked increases in CD68^+^ and Ki67^+^ cells were seen in both Afg09-2990 and Hoti infected mice on day 4 (**Fig 9C**). The level of CD68^+^ cells was increased compared to uninfected mice on day 10 in Hoti infected mice. Collectively, these findings indicated that strain Hoti produces a liver injury similar to that of the lethal strain Afg09-2990. However, livers of Hoti infected mice show evidence of recovery indicated by the presence of EMH and mitotic figures in hepatocytes at later time points.

## Discussion

### MAVS plays a role in CCHFV pathogenesis

This study demonstrates that MAVS activation and proinflammatory cytokine production are important host determinants of severe CCHFV infection. It is not surprising that in the absence of MAVS activity mice were not hypersensitive to CCHFV because IFN-I activity can be induced by multiple redundant pathways, including toll-like receptors. However, it was striking that MAVS deficient mice did not develop severe disease when IFN-I activity was simultaneously blocked. Animals lacking IFN-I are highly susceptible to CCHFV infection [15–20,22]. Therefore, the genetic absence of MAVS and the global blockade of IFN-I activity by antibody should have created an environment highly susceptible to CCHFV infection. To the contrary, despite some weight loss, the absence of MAVS completely protected mice from lethality and liver injury was markedly attenuated. MDA5 deficient mice were not protected from CCHFV, leading us to suspect that RIG-I signaling is the likely sensor upstream of MAVS involved in CCHFV pathogenesis. We did not have access to RIG-I deficient mice to evaluate this model.

Loss of MAVS reduced the viral load in serum and liver compared to WT mice. This was unexpected given that in most systems the loss of MAVS, and consequentially loss of cytoplasmic sensing of RNA viruses, leads to an increase in viral load in the host or enhanced replication in cell culture [33,36–38]. While loss of inflammatory responses clearly contributed to protection, abrogation of cytokine activity alone may not fully explain the decreased viral load. MAVS-dependent inflammation may be critical for CCHFV replication by recruiting inflammatory cells, such as macrophages and neutrophils, to the liver and these cells are important for viral replication and liver injury. Neutrophils have been shown to enhance West Nile virus replication in mice [39]. Monocyte-derived macrophages are highly supportive of CCHFV replication [40]. Accordingly, without infiltration of innate immune cells in MAVS KO mice, CCHFV replication may be limited to Kupffer cells, which are tissue-resident macrophages, thus preventing damage to hepatocytes.

MAVS is also a mediator of liver injury caused by Hepatitis A virus (HAV) in a murine system [38]. However, HAV does not cause a lethal infection in the mouse model, but rather produces a mild and transient liver injury. Our findings expand upon the HAV Hirai-Yuki et al study by demonstrating that loss of MAVS can protect against catastrophic viral infection. Together these data demonstrate that MAVS can be critically involved in the pathogenesis of some viruses, but this is likely the exception given the extent of data showing MAVS is critical for virus control [33,36–38]. The liver is a primary target for both HAV and CCHFV, thus MAVS-driven pathogenesis may be limited to hepatotropic viruses. However, previous studies have shown that mice lacking MAVS in which the IFN-I antibody blockade was also induced, are not protected against Ebola virus, which also targets the liver [29]. Similarly, MAVS-deficient animals were not protected against Rift Valley Fever virus (**S6 Fig)**, another member of the *Bunyavirales* that targets the liver in mice [41]. Therefore, MAVS-dependent pathogenesis in response to viral infection may be more nuanced than simply limited to hepatotropic viruses. Our work and the HAV study highlight the need for further inquiry as to why MAVS may be important in the pathogenesis of certain viruses yet critical for protection against many others.

### Inflammatory cytokines contribute to CCHFV disease severity

Proinflammatory cytokines, such as TNF-α and IL-6, have been implicated as critical factors in the development of severe CCHFV human disease [24–26]. Our findings provide the first experimental evidence that signaling by TNFA-Rs contributes to CCHFV disease progression in the host. Both TNFA-Rs (p55 and p75) appear to contribute to acute CCHFV infection, as mice lacking either or both receptors are equally protected. The default signaling pathway of TNFA-Rs is cell survival in a process regulated by TNF Receptor Associated Factor 2 (TRAF2) [42]. Continuous activation of either of the two TNFA-Rs, such as during aberrant inflammation, can incur a TRAF2 deficiency that disrupts negative regulation of the death domains associated with TNFA-R1, thereby leading to death pathway signaling [43]. We predict that CCHFV replication and extensive TNFA-R activation consumes TRAF2 in both hepatocytes and Kupffer cells, subsequently driving signaling pathways towards cell death. This possibility is consistent with our previous studies demonstrating that CCHFV infected and non-infected bystander cells are lost during liver infection [19]. Curiously, there appears to be a delicate balance in host survival because in some of our studies 100% of TNFA-R KO mice survive and in other studies ~80% of animals survive with a delayed time to death, despite total loss of WT control mice in each study. TNFA-R signaling is critical for liver regeneration, but it can also promote liver injury [44–47]. Therefore, because of the pleiotropic role of TNFA-R signaling in survival and death, it is not surprising that we only observed partial protection that varied between different studies.

Our work identified TNF-α as the principal TNFA-R ligand driving CCHFV pathogenesis. The ability of anti-TNF-α antibodies to protect against CCHFV in a post-exposure setting may have important implications. There are FDA licensed anti-TNF-α antibody therapeutics currently marketed for treating automimmune disorders such as rheumatoid arthritis [48]. Our findings suggest that antibody-based targeting of TNF-α could have therapeutic benefit in humans severely infected with CCHFV. While disrupting IL-6 or IL-1 alone did not prominently prevent CCHFV lethality compared to TNF-α, there was very limited protection. Perhaps blocking these molecules in combination might provide a greater protection in a post-exposure setting. This may explain why MAVS deficient mice were universally protected, whereas TNFA-R knock animals were only partially protected. While our work identified TNF-α as a host target for CCHF, this cytokine is important for immune signaling and altering its levels in the course of disease could be detrimental to the host, particularly if given too early. Indeed, therapies targeting TNF-α for conditions such as sepsis and autoimmune disorders can increase host susceptibility to infection and have a negative outcome [49]. Thus, any use of anti-TNF-α to treat CCHF should be conducted with great caution and perhaps reserved as a salvage therapy in severe cases.

### Differences in induction of inflammatory cytokines by different CCFHV strains can determine disease outcome

CCHFV case fatality rates vary greatly worldwide [3]. CCHFV strains, including AP92 and AP92-like circulating in Greece and Turkey, are associated with a low level of virulence and mortality despite evidence that there is an estimated 6% and 5.2% seropositivity, respectively, in the human population in these areas [50,51]. Despite causing a similar level of liver injury in the mouse system, the non-murine lethal strain Hoti produced a more blunted inflammatory cytokine profile compared to the lethal mouse strain Afg09-2990. These data suggest differences in progression to severe human disease could be related to the ability of specific strains to induce inflammation. Strain Hoti was isolated from a lethal human infection [52] and produces lethal disease in NHPs in some [53], but not all, studies [54]. However the strain consistently produces a less lethal infection in both the IFN-I blockade murine model (this study) and in the IFN-I knockout mice [35] compared to near universal lethality produced by strains Afg09-2990 and strain Ibr-10200 [19,20,28]. This would indicate that species specific differences in host responses to particular strains of CCHFV can impact disease outcome, possibly due to strain differences in host sensing pathways. The fact that stain Hoti causes equivocal levels of liver injury compared to the lethal strain indicates that extrahepatic events are critical for mortality. Strain Afg09-2990 produced higher viremia (**Fig 7C**) and more splenic red pulp necrosis and histocyte infiltration compared to Hoti (**S7 Fig**), which may explain the increased cytokine production. A combination of liver dysfunction resulting in hyperammonia, combined with elevated cytokines including TNF-α, which are known to cause neuronal loss and brain injury [55], could result in a lethal encephalitis. More holistic studies the murine system are warranted to more fully understand how CCHFV causes a fatal infection.

### Study limitations

CCHFV only produces severe disease in rodents when IFN-I signaling is disrupted. Accordingly, CCHFV animal infection studies are generally conducted in IFNAR KO or STAT1 or STAT 2 KO mice or hamsters [15–22] Here, we purposefully avoided using these models because genetic disruption of these systems can result in congenital defects in innate immune signaling [56,57]. For example, IFN-β KO mice have increases in TNF-α levels subsequent to innate immune activation compared to WT animals [58]. To negate the influence of these congenital issues, we used the transient IFN-I antibody blockade CCHFV infection model that we previously developed [19]. Disease produced in this model is similar to that observed in IFNAR KO mice, including the mean time to death [15,20], but with the benefit that IFN-I is only disrupted around the time of infection in otherwise immune intact animals. Also, by using this system we did not have to produce double knockout mice with deficiencies in MAVS or TNFA-R signaling and deletion of the IFNAR, thus avoiding the congenital issues with that background. Nevertheless, IFN-I was blocked in the animals used in our study potentially influencing the findings. While disruption of IFN-I is ostensibly not ideal, some human data indicates that defects in IFN-I activity result in more severe CCHFV infection. Specially, TLR8/9 or TLR3 polymorphisms are associated with acute CCHF [13,14]. Additionally, data from human SARS-CoV-2 infections suggests some of those who develop severe COVID-19 have autoantibodies against various IFN-Is, making them more susceptible to infection [59]. Thus, the antibody blockade model where the IFN-I system is impaired, but not completely absent compared to genetic knockout animals, may more closely emulate conditions that make some humans more susceptible to developing severe CCHF versus other CCHFV rodent systems.

### Host responses to viral infections

Chronic inflammation is a major component of many non-infectious autoimmune diseases, including rheumatoid arthritis (RA), ulcerative colitis (UC), and Crohn’s disease [60,61]. The therapeutic targeting of cytokines, including TNF-α, IL-6 and IL-1, can mitigate disease pathology [60]. Our findings demonstrate that the host response against CCHFV infection contributes to the pathogenic processes supporting an emerging paradigm that host responses to infectious diseases can become driving factors in the pathogenic process. Other work has revealed targeting TNF-α can protect mice against some, but not all, strains of Dengue virus and against hemophagocytic lymphohistiocytosis-like disease induced by infectious disease [36,62], possibly implicating TNF-α as a broad spectrum target for multiple infectious diseases. In addition to TNF-α, emerging evidence suggests that the inflammatory cytokines IL-1 and IL-6 may contribute to lung damage caused by SARS-CoV-2 [63,64]. Collectively, these studies demonstrate that aberrant inflammation produced during viral infections are not stochastic processes, but rather consist of specific molecular events that can be targeted to abate pathogenic effect.

## Methods

### Ethics statement

All animal studies were conducted in compliance with the Animal Welfare Act and other federal statutes and regulations relating to animals and experiments involving animals and adheres to principles state in the Guide for the Care and Use of Laboratory Animals, National Research Council [65]. Mouse work was granted approval by the United States Army Medical Research Institute for Infectious Diseases (USAMRIID) Animal Care and Use Committee. The facilities where this research was conducted are fully accredited by the Association for Assessment and Accreditation of Laboratory Animal Care International. Animals were scored daily for signs of disease (including ruffed fur, decreased mobility/lethargy, and limited response with stimulation and reduced interaction with peers) and were humanely euthanized when pre-established scoring conditions were met.

### Viruses and cells

Huh7 and SW13 cells were propagated in Dulbecco’s Modified Eagles Medium with Earle’s Salts (DMEM) (Corning) supplemented with 10% fetal bovine serum (FBS) (Gibco) 1% Penicillin/Streptomycin (Gibco), 1% Sodium Pyruvate (Sigma), and 1% L-Glutamine (HyClone) and 1% HEPES (Gibco). Minimally passaged CCHFV strain Afg09-2990 [66] was passaged three times in Huh7 cells. Strain Kosovo Hoti (Hoti) [52] was kindly provided by Dr. Tatjana Avšič - Županc (University of Ljubljana) and Dr. Heinz Feldmann and had been passaged 7 times in Vero E6 cells and 3 times in SW13 cells prior to receipt at USAMRIID, it was then amplified by one passage in Huh7 cells. Viruses were collected from clarified cell culture supernatants and stored at -80°C. All CCHFV work was handled in a BSL-4 containment laboratory at USAMRIID that was fully compliant with applicable federal statutes.

### Mice

C57BL/6J (BL6), B6.129, MAVS KO (B6;129-Mavstm1Zjc/J), MDA5 KO (B6.Cg-Ifih1tm1.1Cln/J), TNF-α KO (B6;129S-Tnftm1Gkl/J), IL-6 KO (B6.129S2-Il6tm1Kopf/J), TNFAR DBL KO (B6.129S-Tnfrsf1atm1Imx Tnfrsf1btm1Imx/J), p55 KO (C57BL/6-(Tnfrsf1atm1Imx/J), p75 KO (B6.129S7-Tnfrsf1btm1Imx/J) and Rag2 KO (B6.Cg-Rag2tm1.1Cgn/J) mice (6–8 weeks old) were purchased from The Jackson Laboratory. Mice were challenged with 100 PFU of the indicated CCHFV strain by IP injection of virus diluted in a total volume of 0.2 ml Phosphate buffered saline (PBS). To induce the IFN-I antibody blockade, mice were injected by the IP route with 2.5 mg of anti-IFNR1 (MAb-5A3) (Leinco Technologies, Inc) diluted in PBS 24 h post-virus exposure as previous described [19].

### Plaque assay

Liver homogenates were diluted 1:10 in EMEM and subsequently, 100 μl of sample was adsorbed to confluent SW13 cell monolayers in 6-well plates for 1 h in a 37°C 5% CO_2_ incubator and rocked every ~15 m. Following adsorption, a 2 ml solid overlay (Earle’s basal minimal essential medium (EBME), 0.5% agarose, 5% heat-inactivated FBS, antibiotics (100 U/ml penicillin, 100 μg/ml of streptomycin, and 50 μg/ml of gentamicin) was added to each well. Plates were incubated for three days in a 37°C 5% CO_2_ incubator and stained with 2 ml of solid overlay mixture containing 5% neutral red (Gibco). Cells were incubated an additional 24 h in a 37°C 5% CO_2_ incubator before plaque counting. Virus titer was calculated per gram of tissue.

### RT-qPCR

Mouse serum samples were inactivated using a 3:1 ratio of TRIzol LS Reagent to serum (ThermoFisher, Waltham, MA). Liver tissue was homogenized in 750 μl DMEM using a Tissuelyser II (Qiagen, Germantown, MD). Supernatants were inactivated using a 3:1 ratio of TRIzol LS to supernatant and placed at -80°C. Total nucleic acid was purified using the EZ1 Virus Mini Kit v 2.0 (Qiagen) using the EZ1 Advanced XL robot (Qiagen) according to the manufacturer’s recommendations. Viral load was determined using a real-time RT-PCR assay specific to CCHFV [67].

### Antibody-mediated TNF blockade

Mice were treated with 2.0 mg per dose of anti-TNF-α murine antibody XT3.11 (Bio-X-Cell) at the indicated time points by the IP route. An isotype antibody HRP (Bio-X-cell) was used as a control.

### Histology

Necropsy was performed and tissues were immersed in 10% neutral buffered formalin for 30–70 days. Tissue were then trimmed and processed according to standard protocols [68]. Histology sections were cut at 5–6 μm on a rotary microtome, mounted onto glass slides and stained with hematoxylin and eosin (H&E). Examination of the tissue was performed by a board-certified veterinary pathologist.

### Cytokine and chemokine analysis

Serum cytokine and chemokine analysis was performed using a magnetic bead-based plex mouse panel (ThermoFisher) targeting the indicated molecules. 25 μl of serum per mouse per time point was used. Plates were analyzed on a MAGPIX system (Luminex) and quantitated against standard curves using xPonent 4.2 for MAGPIX (Luminex) software. Alternatively, TNF-α levels in liver homogenates (25 μl) were detected using a commercially available ELISA (R&D systems; Minneapolis, MN) following the manufacturer’s protocol.

### Liver enzymes

Aspartate aminotransferase (AST) and alanine aminotransferase (ALT) levels were measured in serum from CCHFV infected animals using a Piccolo Xpress (Abcam) and a general chemistry 13 panel following the manufacturer’s protocol.

### In situ hybridization

CCHFV were detected in infected liver samples by ISH probes targeting M-segment of CCHFV strain Afg09-2990 (reverse complement strand of nucleotides positions 631-2702HM452306.1) or strain Hoti (reverse complement sequence of 761–2698 bp of MH483985.1) (Advanced Cell Diagnostics; Newark, CA). Formalin-fixed paraffin embedded (FFPE) liver sections were deparaffinized and peroxidase blocked. Sections were then incubated with ISH probes at 40°C for 2 h, rinsed and the signal amplified by applying Pre-amplifier and Amplifier conjugated with HRP. A red substrate-chromogen solution was applied for 10 m at ambient temperature. The slides were further stained with hematoxylin. Images were captured on a Zeiss LSM 880 confocal system and processed using ImageJ software.

### NanoString gene expression analysis

Total RNA samples were analyzed using the nCounter Mouse Inflammation v2 panels as previously reported [19]. Probe set-target RNA hybridization reactions were performed according to the manufacturer’s protocol. For each hybridization reaction, 100 ng total RNA was used or any quantity that was present in a 5-μl aliquot of purified RNA if less than 100 ng. Purified probeset-target RNA complexes from each reaction were processed and immobilized on nCounter Cartridges using an nCounter *MAX* Prep Station and transcripts were quantified on the Digital Analyzer (GEN 2). Data from each NanoString panel were first processed independently using nSolver 4.0 software (NanoString) as follows: following quality control checks on the individual RCC files, raw counts across samples were normalized to the geometric mean counts of spiked synthetic DNA positive controls present in the hybridization reactions to mitigate platform-associated sources of variation. No background subtraction or thresholding was performed at this stage. Candidate reference genes were selected using the nCounter Advanced Analysis (nCAA) module (version 2.0.115), which implements the geNorm algorithm for downselection. Starting with a set of six candidate reference genes (*Cltc*, *Gusb*, *Hprt*, *Pgk1*, *Gapdh*). For each sample, normalization was performed by dividing counts for each gene by the geometric mean of the five selected reference genes. These two normalized data sets were then combined in nSolver as a multi-RLF merge experiment, and then input to the nCAA module for differential expression, gene set, and biological pathway analysis. The threshold for differential expression was [log2 fold-change] > 1 and a P value < 0.05.

### IFA of tissues

Formalin-fixed paraffin embedded (FFPE) tissue sections were deparaffinized using xylene and a series of ethanol washes. The sections were heated in Tris-EDTA buffer (10mM Tris Base, 1mM EDTA Solution, 0.05% Tween 20, pH 9.0) for 15 m to reverse formaldehyde crosslinks. After rinses with PBS (pH 7.4), the section were blocked with PBT (PBS +0.1% Tween-20) containing 5% normal goat serum or PBS with 5% bovine serum albumn (CLEC4F staining) overnight at 4°C. Then the sections were incubated with primary antibodies: rabbit polyclonal anti-myeloperoxidase (MPO) at a dilution of 1:200 (A039829-2, Dako Agilent Pathology Solutions, Carpinteria, CA, USA), rat monoclonal anti-CD45 antibody at a dilution of 1:100 (05–1416, Millipore Sigma, Burlington, MA, USA), rabbit polyclonal anti-CD68 at a dilution of 1:200 (ab125212, Abcam, Cambridge, MA, USA), and mouse monoclonal anti-Ki67 at a dilution of 1:200 (clone B56, BD Biosciences, San Jose, CA, USA) for 2 h at room temperature. After rinses with PBT, the sections were incubated with secondary goat anti-rabbit or anti-chicken Alexa Fluor 488 at dilution of 1:500 (ThermoFisher) and goat anti-mouse or anti-rat Alexa Fluor 568 at a dilution of 1:500 (ThermoFisher) antibodies, for 1 hour at room temperature. For CLEC4F and CCHFV N protein detection, samples were incubated with a polyclonal goat anti-CLEC4F antibody at 1:20 dilution (PA5-47396; ThermoFisher) and the anti-CCHFV N murine monoclonal antibody MAb-9D5 protein at 1:500 dilution overnight at 4°C. Following primary antibody incubation, PBS washed sections were incubated with Alexa Fluor 488-conjugated goat anti-mouse IgG antibody and Alexa Fluor 561-conjugated anti-goat IgG antibody at 1:1000 dilution for 1 h at room temperature. Sections were cover slipped using the Prolong Diamond mounting medium with DAPI (ThermoFisher). Images were captured on a Zeiss LSM 880 or LSM700 (CLEC4F staining) confocal system (Zeiss, Oberkochen, Germany) processed using ImageJ software (National Institutes of Health, Bethesda, MD).

### Statistical analysis

Significance of weight loss, cytokines, blood chemistry and viral load was determined using ANOVA with the Bonferroni correction. Survival statistics utilized the log-rank test. Significance levels were set at a *p* value less than 0.05. All analyses were performed using GraphPad Prism 9.1.2 software (GraphPad Software, San Diego, CA).

## Supporting information

S1 Fig CCHFV infection of MAVS KO mice in presence of IFN-I blockade. C57BL/6 WT mice (BL6) or MAVS KO mice (n = 5 per group) were infected with CCHFV and survival and weight loss were monitored and plotted using Prism software. All mice were treated with mAb-5A3 24h after infection to block IFN-I. Significance determined by log-rank analysis; ***p<0.0001.
(TIF)

S2 Fig Pathway activation comparison from CCHFV infected WT and MAVS KO mice. Gene transcript data obtained from NanoString analysis was input into Ingenuity Pathway Analysis software (QIAGEN) for analysis of pathway activation/inactivation during CCHFV infection. CCHFV infection in WT mice (day 4) showed significant increases in several immune pathways over MAVS KO mice. Additionally, several cell signaling pathways were downregulated during CCHFV infection in WT mice (day 4). By 10 DPI in MAVS KO mice, these changes in expression were more similar compared to WT day 4 but still significantly different.
(TIF)

S3 Fig CCHFV infection of TNFA-R DBL KO mouse livers. A. Representative H&E staining of TNFA-R DBL KO mice on day 10 showing mitotic figures (arrows), indicative of liver recovery/regenerative response. B. Representative liver section from a day 10 infected TNF-R DBL KO mouse were stained with anti-CLEC4F (red) and anti-CCHFV N protein (green) antibodies. Cell nuclei were stained with DAPI
(TIF)

S4 Fig Rag2 mice infected with CCHFV strain Afg09-2990 and Hoti. A. Rag2-deficient mice (n = 8/group) were infected with 100 PFU of strain Afg09-2990 or strain Hoti by the IP route and treated with anti-5A3 for the IFN-I blockade 24 h after challenge. Survival was monitored for 20 days. Statistical significance was determined by log-rank; **p<0.005. B. TNF-α ELISA from serum of infected mice on day 4 (Afg09-2990 n = 3, Hoti; n = 2;) or uninfected mice (n = 2). Statistical significance determined by One-way ANOVA **p<0.005, *p<0.05. C. Serum ALT and AST concentrations in the indicated infected mice (n = 3) on day 4 post-infection or uninfected mice (n = 2). The gray area shows the normal levels in mice.
(TIF)

S5 Fig Liver histopathology on day 10 and 15 in Hoti infected mice. A&B. Liver H&E of Hoti infected mice showing inflammation in the liver of a day 10 animal. Higher magnification (B; 20x) shows that the inflammation is predominantly mononuclear (compared the neutrophilic inflammation at day 4). Note the presence of mitotic figures (arrows) and an apoptotic hepatocyte (arrowhead). C&D. Day 15 Hoti infected mice have decreased inflammation compared to the day 10 animals. Some mononuclear inflammation surrounding a bile duct in a portal area with minimal infiltrates in the adjacent hepatic parenchyma was observed. Note the absence of mitotic figures and more uniform presence of glycogen as seen in a normal liver.
(TIF)

S6 Fig Infection of Non-Tg and MAVS KO mice with Rift Valley Fever virus. Non-Tg and MAVS KO mice (n = 8 per group) were infected with 100 pfu of Rift Valley Fever virus [69] survival and weight loss were monitored and plotted using Prism software. NS: not significant (log-rank).
(TIF)

S7 Fig Histopathological lesions of the spleen in Afg09-2990 and Hoti infected WT mice on day 4. **A**. H&E staining showing depletion of lymphocytes in the white pulp and increased cell density in the red pulp of Afg09-2990 infected mice. Neutrophilic inflammation in the red pulp (black arrows), increased cell density in the red pulp due to histiocytes infiltration and decreased cellularity of the white pulp with tingible body macrophages (lymphocyte apoptosis) was seen in both groups. Hoti infected mice had slightly less tinglible bodies and fewer histiocytes infiltration in the red pulp. Uninfected animals had a normal spleen. **B**. Pathology score for the indicated lesions. Statistical significant denoted by *p<0.05 (T-test).
(TIF)
