# Supplementary figures and images for: The host inflammatory response contributes to disease severity in Crimean-Congo hemorrhagic fever virus infected mice
Source: PLoS Pathog. 2022 May 19;18(5):e1010485. doi: 10.1371/journal.ppat.1010485 (PMC9119488; doi:10.1371/journal.ppat.1010485)

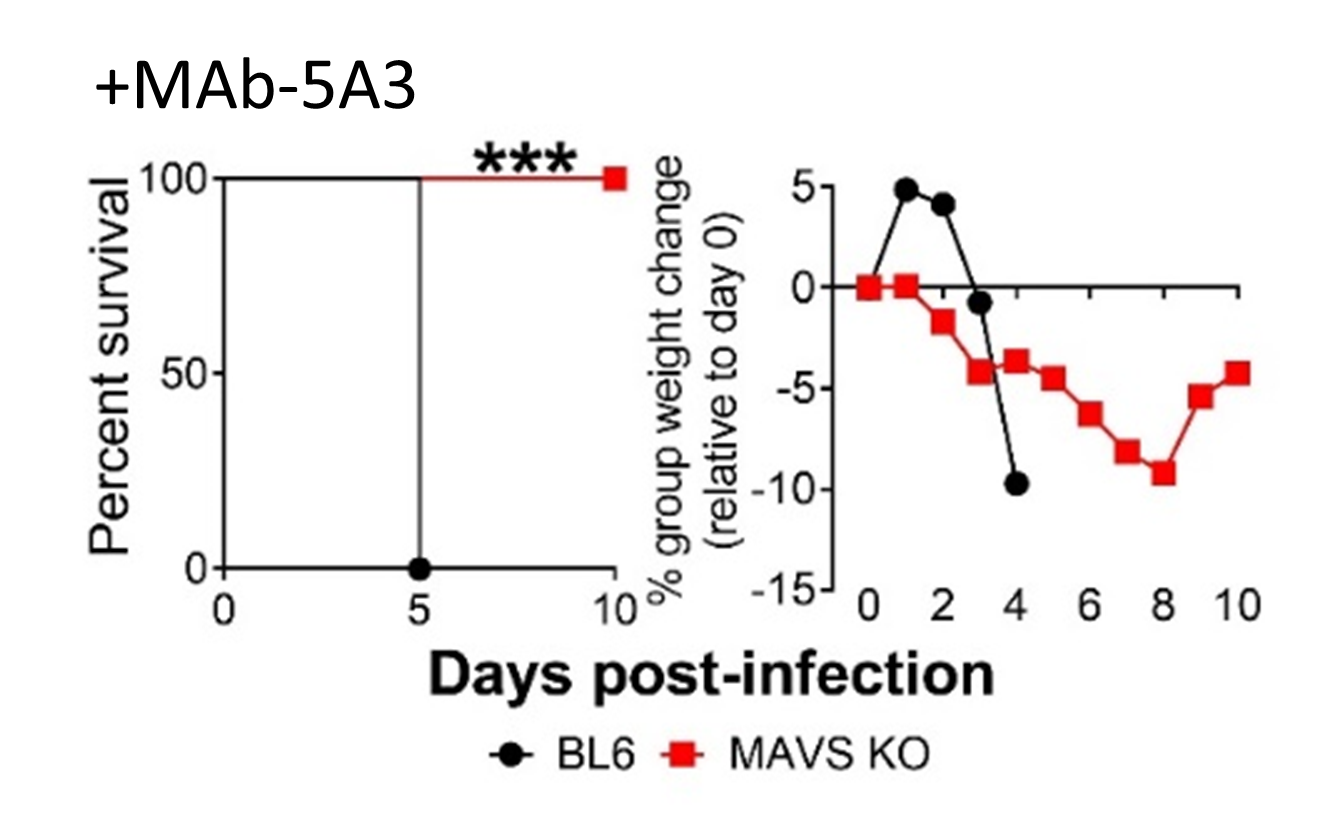

Supplement: S1 Fig — C57BL/6 WT mice (BL6) or MAVS KO mice (n = 5 per group) were infected with CCHFV and survival and weight loss were monitored and plotted using Prism software. All mice were treated with mAb-5A3 24h after infection to block IFN-I. Significance determined by log-rank analysis; ***p<0.0001. (TIF) [file ppat.1010485.s001.TIF]

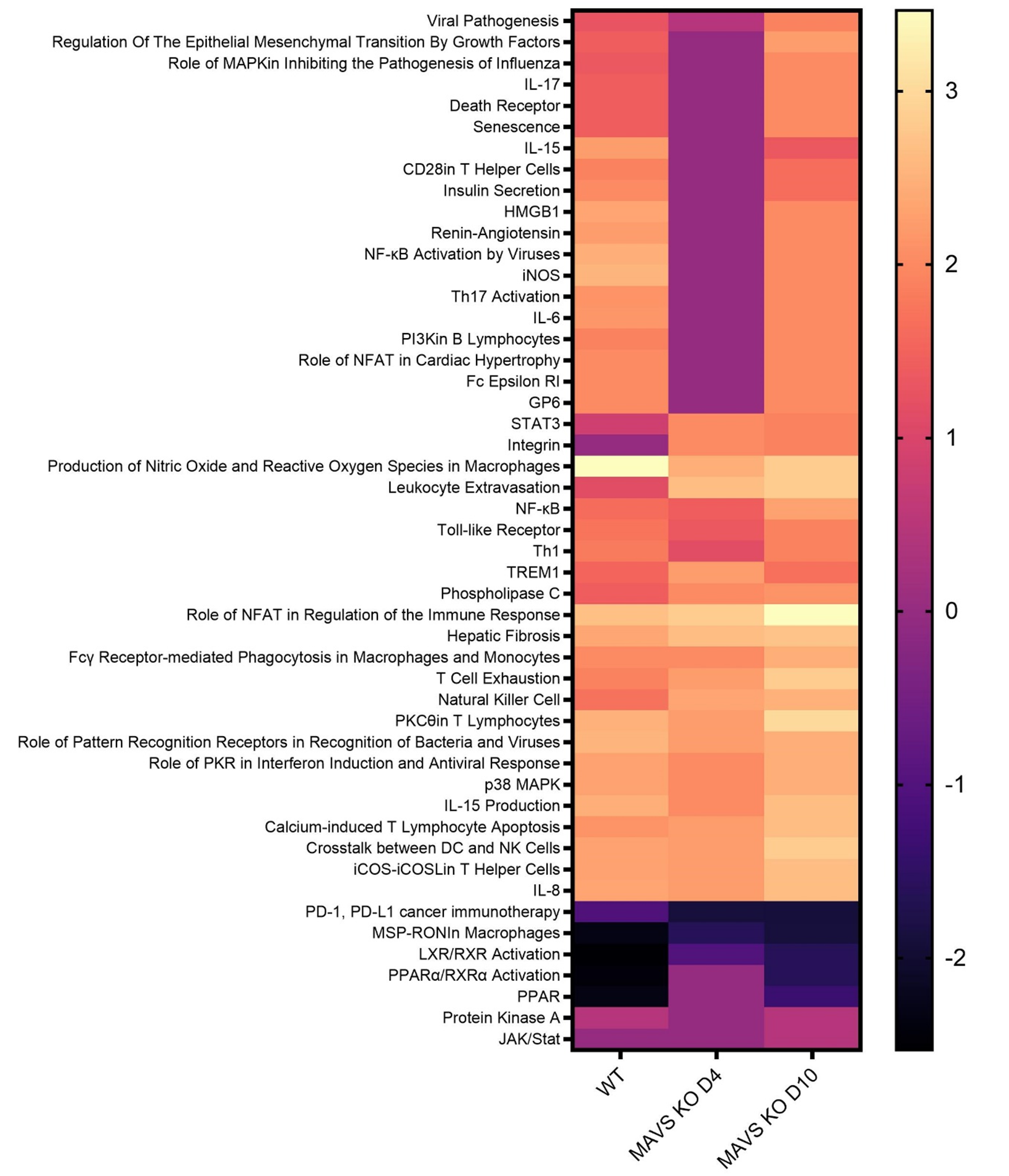

Supplement: S2 Fig — Gene transcript data obtained from NanoString analysis was input into Ingenuity Pathway Analysis software (QIAGEN) for analysis of pathway activation/inactivation during CCHFV infection. CCHFV infection in WT mice (day 4) showed significant increases in several immune pathways over MAVS KO mice. Additionally, several cell signaling pathways were downregulated during CCHFV infection in WT mice (day 4). By 10 DPI in MAVS KO mice, these changes in expression were more similar compared to WT day 4 but still significantly different. (TIF) [file ppat.1010485.s002.TIF]

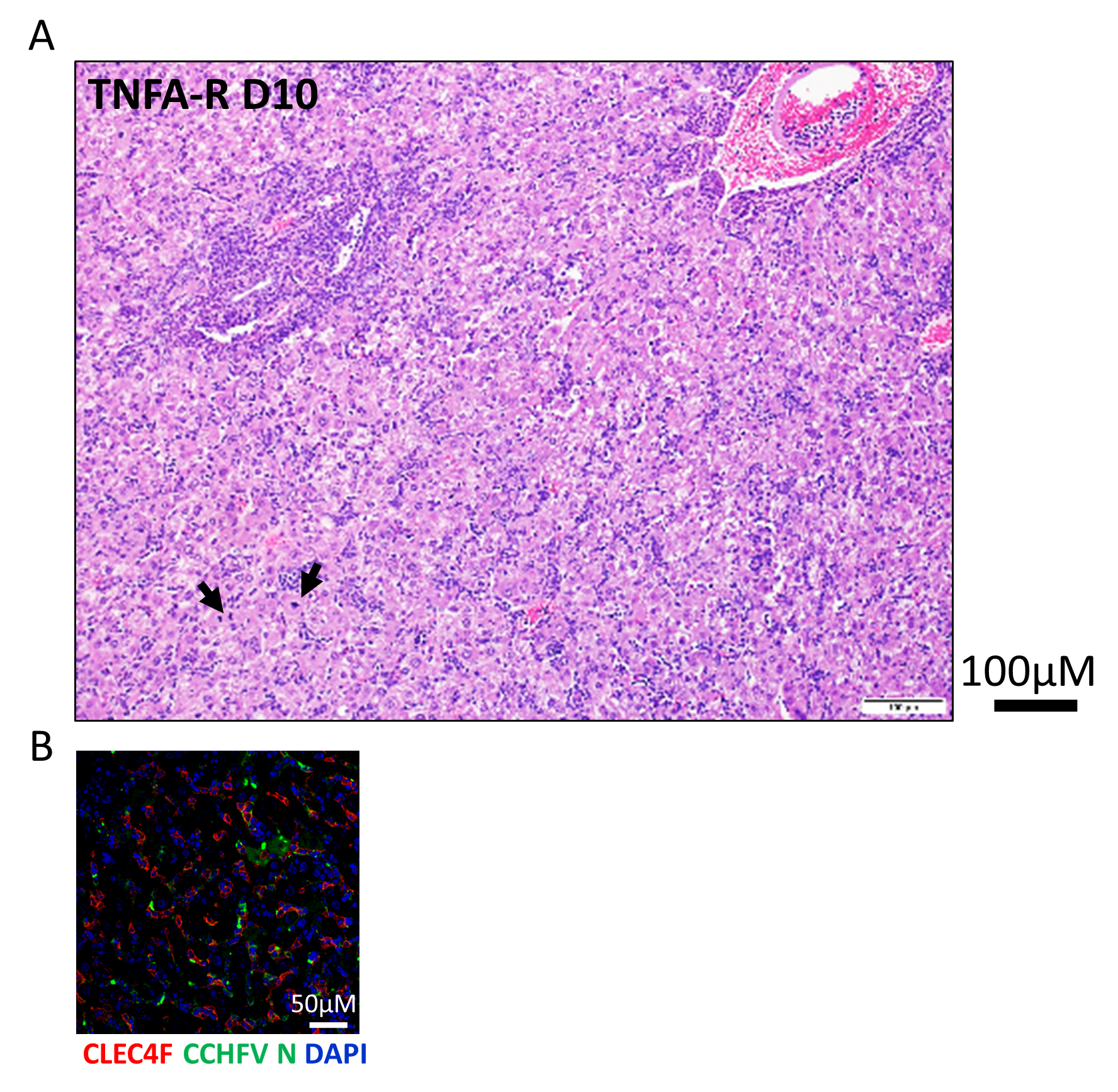

Supplement: S3 Fig — A. Representative H&E staining of TNFA-R DBL KO mice on day 10 showing mitotic figures (arrows), indicative of liver recovery/regenerative response. B. Representative liver section from a day 10 infected TNF-R DBL KO mouse were stained with anti-CLEC4F (red) and anti-CCHFV N protein (green) antibodies. Cell nuclei were stained with DAPI (TIF) [file ppat.1010485.s003.TIF]

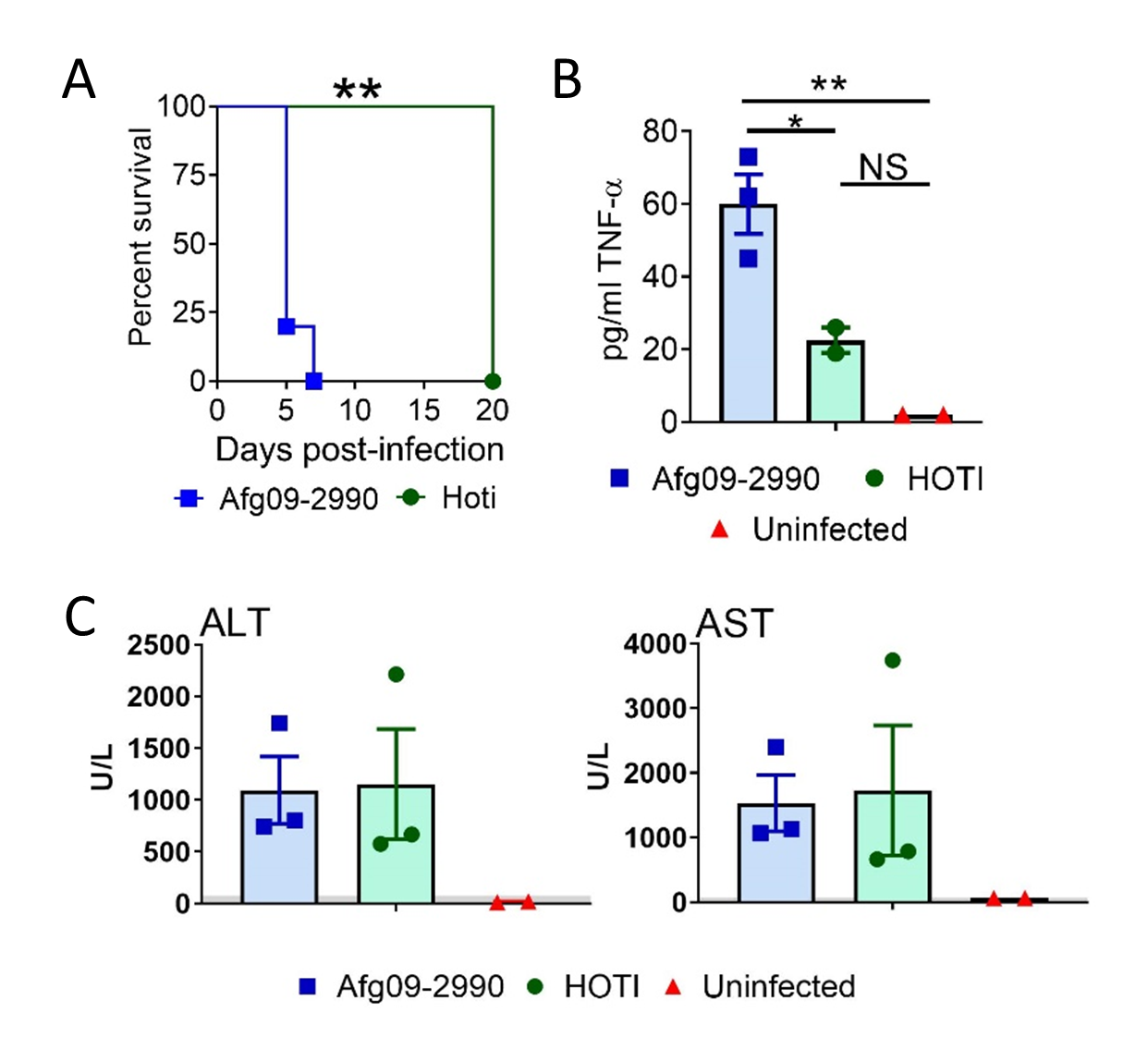

Supplement: S4 Fig — A. Rag2-deficient mice (n = 8/group) were infected with 100 PFU of strain Afg09-2990 or strain Hoti by the IP route and treated with anti-5A3 for the IFN-I blockade 24 h after challenge. Survival was monitored for 20 days. Statistical significance was determined by log-rank; **p<0.005. B. TNF-α ELISA from serum of infected mice on day 4 (Afg09-2990 n = 3, Hoti; n = 2;) or uninfected mice (n = 2). Statistical significance determined by One-way ANOVA **p<0.005, *p<0.05. C. Serum ALT and AST concentrations in the indicated infected mice (n = 3) on day 4 post-infection or uninfected mice (n = 2). The gray area shows the normal levels in mice. (TIF) [file ppat.1010485.s004.TIF]

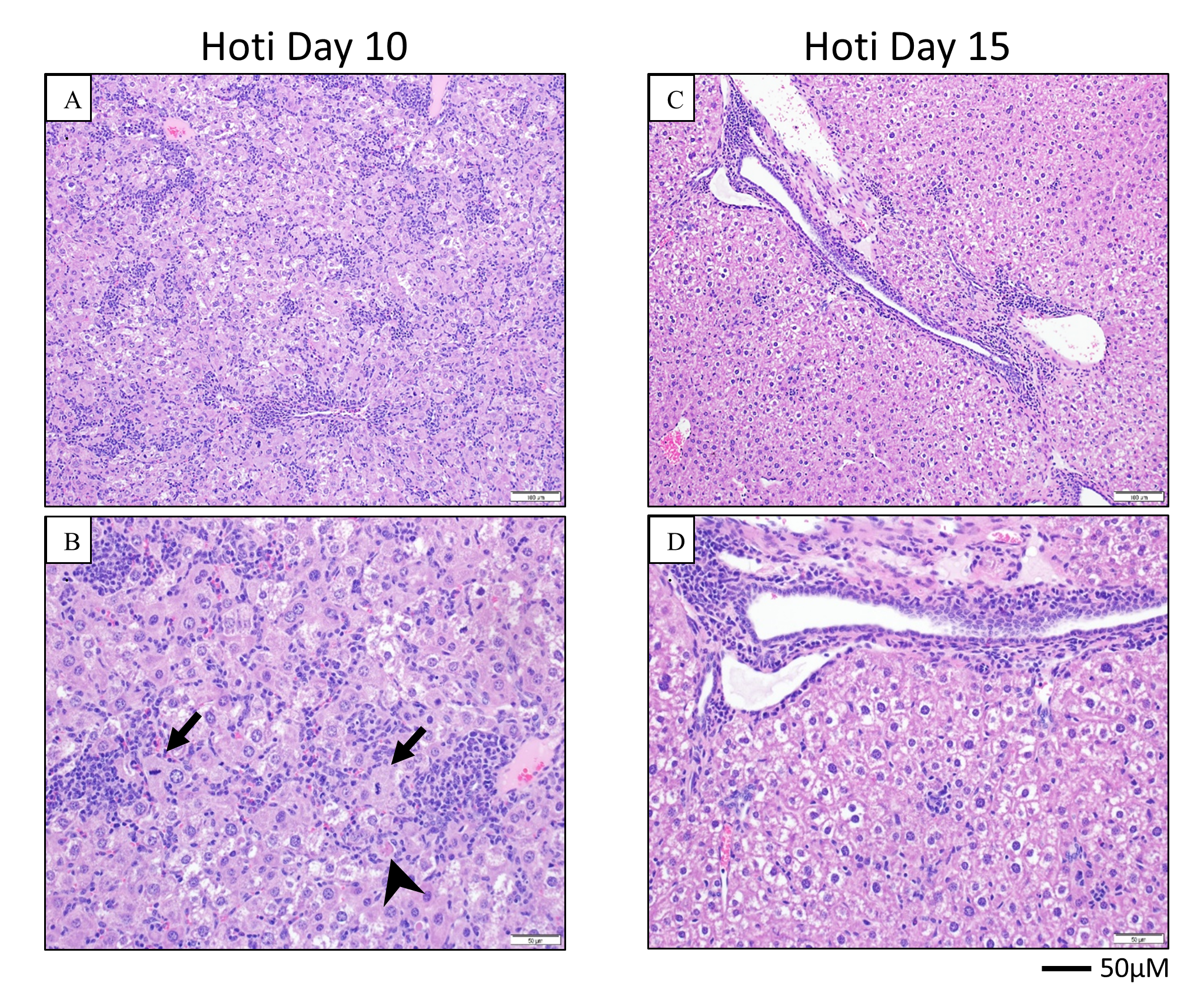

Supplement: S5 Fig — A&B. Liver H&E of Hoti infected mice showing inflammation in the liver of a day 10 animal. Higher magnification (B; 20x) shows that the inflammation is predominantly mononuclear (compared the neutrophilic inflammation at day 4). Note the presence of mitotic figures (arrows) and an apoptotic hepatocyte (arrowhead). C&D. Day 15 Hoti infected mice have decreased inflammation compared to the day 10 animals. Some mononuclear inflammation surrounding a bile duct in a portal area with minimal infiltrates in the adjacent hepatic parenchyma was observed. Note the absence of mitotic figures and more uniform presence of glycogen as seen in a normal liver. (TIF) [file ppat.1010485.s005.TIF]

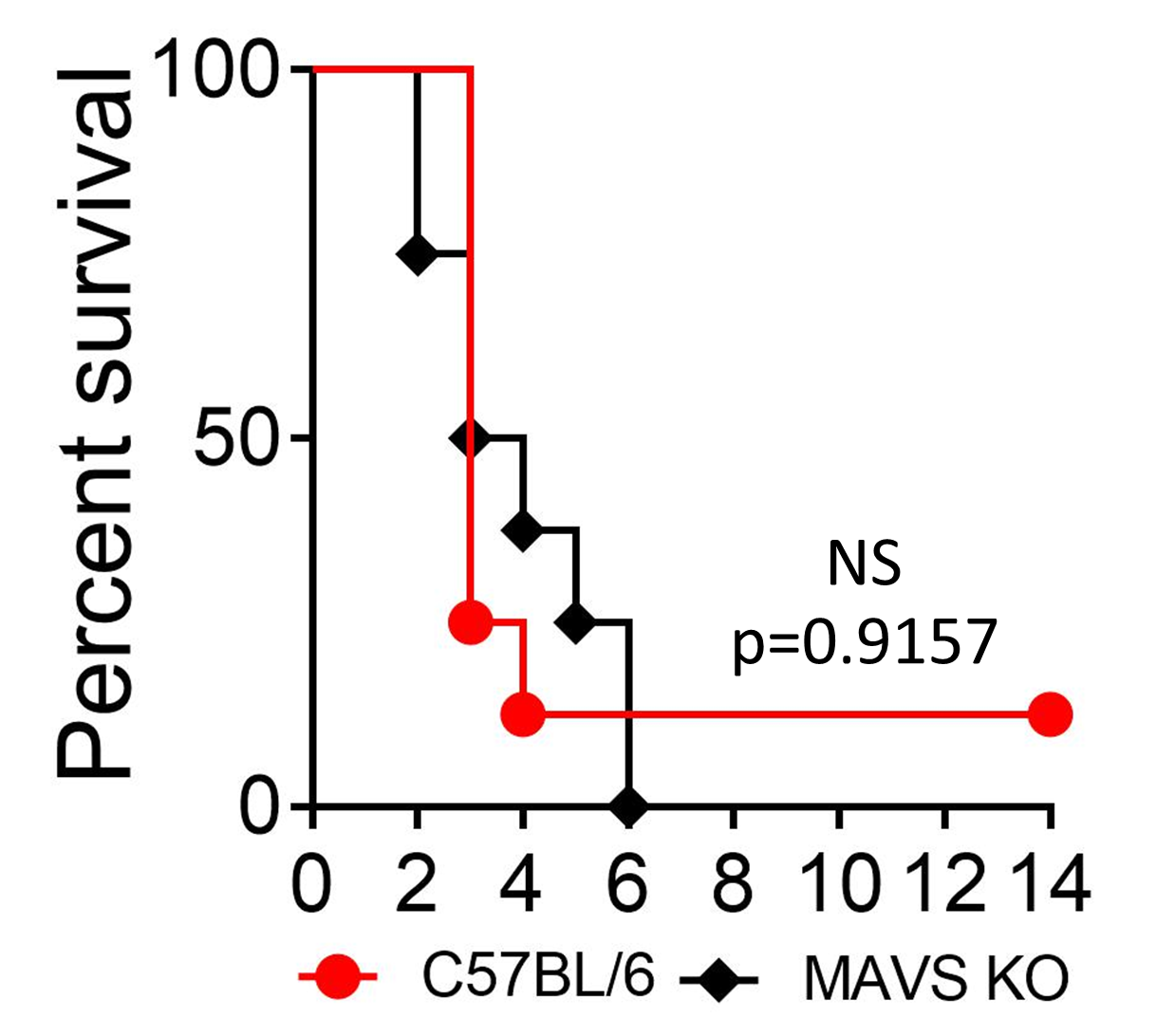

Supplement: S6 Fig — Non-Tg and MAVS KO mice (n = 8 per group) were infected with 100 pfu of Rift Valley Fever virus [69] survival and weight loss were monitored and plotted using Prism software. NS: not significant (log-rank). (TIF) [file ppat.1010485.s006.TIF]

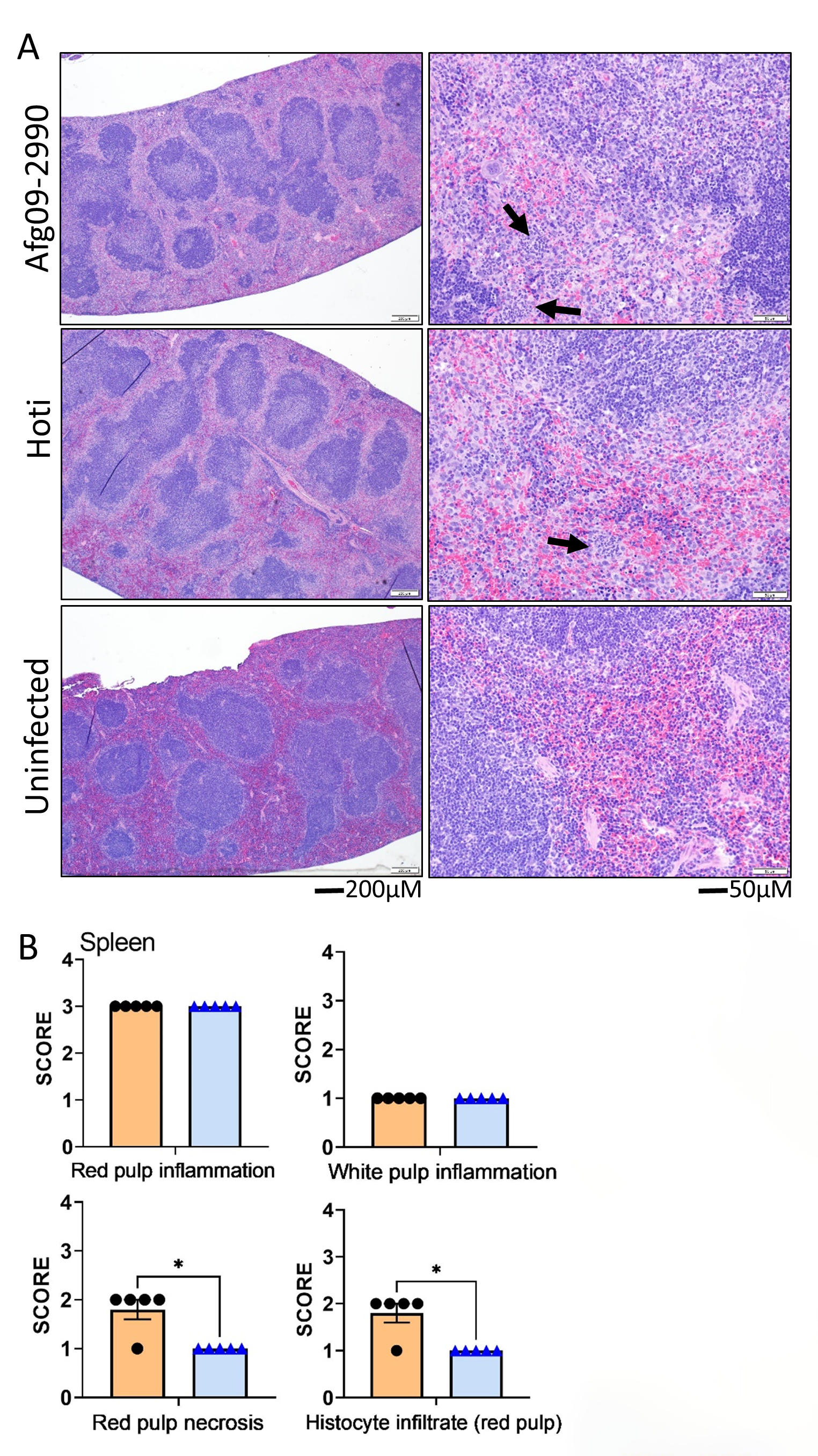

Supplement: S7 Fig — A. H&E staining showing depletion of lymphocytes in the white pulp and increased cell density in the red pulp of Afg09-2990 infected mice. Neutrophilic inflammation in the red pulp (black arrows), increased cell density in the red pulp due to histiocytes infiltration and decreased cellularity of the white pulp with tingible body macrophages (lymphocyte apoptosis) was seen in both groups. Hoti infected mice had slightly less tinglible bodies and fewer histiocytes infiltration in the red pulp. Uninfected animals had a normal spleen. B. Pathology score for the indicated lesions. Statistical significant denoted by *p<0.05 (T-test). (TIF) [file ppat.1010485.s007.TIF]
